# Novel MAFG-METTL14-SCD1 axis regulates lipid metabolism mediating choroidal melanoma distant metastasis

**DOI:** 10.1186/s13046-025-03595-1

**Published:** 2025-11-29

**Authors:** Xi Zhang, Xiaoyun Hu, Chen Fu, Peng Yuan, Yan Yang, Jiling Ru, Yingqi Zhao, Xianglong Zhu, Xiaonan Zhang, Xianjie Liu, Li Han, Jun Li, Xue Bai, Zhe Zhang, Hong Ning, Huizhe Wu, Minjie Wei

**Affiliations:** 1 Department of Pharmacology, School of Pharmacy, China Medical University, Shenyang, Liaoning Province P. R. China; 2 Liaoning Key Laboratory of molecular targeted anti-tumor drug development and evaluation, Liaoning Cancer immune peptide drug Engineering Technology Research Center, China Medical University, Shenyang, Liaoning Province P. R. China; 3 Scientific experimental center, School of Pharmacy, China Medical University, Shenyang, Liaoning Province P. R. China; 4 Department of General surgery, The Fourth Affiliated Hospital of China Medical University, Shenyang, Liaoning Province P. R. China; 5 Department of Gastroenterology, The Fourth Affiliated Hospital of China Medical University, Shenyang, Liaoning Province P. R. China; 6 Department of Ophthalmology, The First Hospital of China Medical University, Shenyang, Liaoning Province P. R. China; 7 Department of Urology, The First Hospital of China Medical University, Shenyang, Liaoning Province P. R. China

**Keywords:** Choroidal melanoma, Metastasis, Lipid metabolism, High-fat diet, SCD1

## Abstract

**Background:**

Tumor invasion and metastasis are strongly influenced by cell membrane fluidity, regulated by lipid metabolism. In choroidal melanoma (CM), a highly metastatic cancer, the relationship between lipid metabolism, membrane fluidity, and metastatic mechanisms remains unclear.

**Methods:**

We examined m^6^A methylation in CM patient samples. Lipidomic profiling was performed in control, METTL14-silenced, or SCD1-silenced CM cells. Transcriptomics were analyzed after METTL14 manipulation. Transmission electron microscopy assessed ultrastructural changes, while multiplex immunohistochemistry validated the clinical relevance of the MAFG–METTL14–SCD1 axis. The anti-metastatic effect of combining the SCD1 inhibitor aramchol with a stearate-rich diet (S-HFD) was tested in nude mouse CM metastasis models.

**Results:**

Lipidomics revealed that SCD1 promotes CM progression via cardiolipin and fatty acid metabolism pathways. Silencing SCD1 reduced membrane fluidity, while its upregulation in CM was driven by METTL14-mediated m^6^A methylation at the 2492 mRNA site. Elevated MAFG expression further activated METTL14. Mechanistically, this MAFG–METTL14–SCD1 axis enhanced CM invasiveness. In preclinical models, aramchol combined with S-HFD markedly suppressed distant metastasis.

**Conclusions:**

Our study identifies SCD1-mediated lipid remodeling as a key driver of enhanced membrane fluidity and metastatic potential in CM. Inhibition of SCD1 increases lipid saturation, reduces membrane fluidity, induces oxidative stress, and suppresses liver and lung metastasis. The MAFG–METTL14–SCD1 axis thus represents a critical regulator of CM progression, and combined therapeutic targeting with aramchol and S-HFD offers promising translational potential.

**Supplementary Information:**

The online version contains supplementary material available at 10.1186/s13046-025-03595-1.

## Background

 Uveal melanoma (UM) stands as the predominant primary intraocular malignancy in adults, with over 90% of cases localized to the choroid [1]. Globally, close-range radiotherapy and enucleation remain the primary treatments for choroidal melanoma (CM) [2, 3]. However, enucleation can lead to significant disability and severe patient harm, as evidenced by the fact that 50% of patients who undergo this procedure experience distant metastasis after primary tumor treatment, a major contributor to mortality in CM [4, 5]. Therefore, understanding the mechanisms underlying CM metastasis is crucial not only to prevent tumor dissemination but also to improve the prognosis of affected individuals.

Tumor invasion and metastasis are heavily influenced by alterations in cell membrane fluidity, with phospholipids serving a central function in maintaining membrane structure and supporting critical cellular functions such as growth, differentiation, and motility [6–8]. The relationship between lipid metabolism, membrane fluidity, and tumor progression warrants further exploration, as lipid metabolism is intricately tied to tumor invasion and spread. Lipid metabolism provides energy to tumor cells and contributes essential components for membrane synthesis, as well as raw materials for signaling molecules involved in metastasis [9, 10]. Disruptions in lipid metabolism lead to changes in membrane lipid composition, thereby impacting membrane viscosity and fluidity [11, 12]. For example, Mao et al. demonstrated that the transcription factor EB (TFEB) activates Estrogen-related receptor α (ERRα), which increases the unsaturation of fatty acyl groups in glycerophospholipids (GPs), enhancing membrane fluidity and promoting invasion and metastasis in endometrial cancer (EC) [13]. Additionally, Liu et al. showed that lysophosphatidylcholine acyltransferase 1 (LPCAT1) regulates the synthesis of saturated fatty acyl phosphatidylcholine (SFA-PC) and monounsaturated fatty acids (MUFA), influencing cell membrane fluidity [14]. Although substantial evidence implicates lipid metabolism in melanoma pathogenesis, the link between lipid metabolism and membrane fluidity in CM, as well as the mechanisms by which lipid metabolism influences membrane fluidity to drive invasion and metastasis, remains poorly understood [15, 16]. Stearoyl-CoA desaturase 1 (SCD1), an essential catalyst in lipid processing, controlling MUFA synthesis. It facilitates the conversion of saturated fatty acids (SFAs), particularly palmitoyl-CoA (16:0) and stearoyl-CoA (18:0), into palmitoleic acid (16:1) and oleic acid (18:1), respectively [17]. Alterations in SCD1 within tumors can disrupt MUFA levels, disturbing the balance between MUFA and saturated fatty acids, thus affecting cell lipid composition and membrane fluidity [18, 19]. In hepatocellular carcinoma (HCC), downregulation of SCD1 expression or activity alters the lipid profile of tumor cells, impairing membrane fluidity and inhibiting intratumoral metastasis [20]. In colorectal cancer (CC), SULT2B1 interacts with SCD1 to modulate lipid metabolism, facilitating tumor metastasis [21]. The impact of combining SCD1 inhibitor with specific dietary regimens on CM metastatic remains unclear and warrants further investigation.

Lipid metabolism is regulated by multiple mechanisms, including epigenetic control, high-fat diet (HFD) exposure, AMPK signaling, and PPAR transcription factors, with a particular emphasis on epigenetic regulation [22–25]. Among these, the N6-methyladenosine (m^6^A) modification, a prevalent RNA epigenetic alteration, exerts considerable influence on tumor development and spread by modulating the expression of oncogenes and tumor suppressor genes, thus either facilitating or suppressing tumor progression, particularly in the context of CM metastasis [26–30]. Notably, m^6^A modification is closely linked to metabolic processes, prompting considerable research interest [31–34]. For instance, Peng et al. demonstrated that METTL5 enhances de novo lipogenesis in HCC cells, increasing free fatty acid levels and stimulating fatty acid metabolism and tumorigenesis in vivo. They showed that METTL5-mediated m^6^A modification of 18 S rRNA enhances the translation of mRNAs linked to fatty acid metabolism, highlighting the dual role of the METTL5-ACSL axis in lipogenesis and fatty acid oxidation to drive HCC progression [35]. Similarly, Chen et al. discovered that FTO influences the levels of sterol regulatory element-binding proteins (SREBPs) and cell death-inducing DFF45-like effector c (CIDEC) within HCC cells. In particular, FTO’s m^6^A removal activity facilitates SREBP1c mRNA transport to the nucleus and its subsequent maturation, leading to enhanced expression and CIDEC activation, which results in increased lipid production and storage in HCC cells [36]. However, the regulation of the key lipid-metabolism enzyme SCD1 in CM has not yet been reported.

In summary, this study focuses on valuable CM pathological tissues as a critical entry point. By integrating transcriptomics, lipidomics, clinical data, and comprehensive in vivo experiments, this investigation seeks to reveal the molecular pathways by which lipid metabolism in CM influences membrane fluidity, thereby driving invasion and metastasis. Comprehensive multi-omics analysis elucidated the critical interplay between lipid metabolism, cell membrane fluidity, invasion and metastasis, confirming the essential roles of these processes in tumor development and proposing a novel regulatory paradigm. Preclinical mouse models suggest that therapeutic interventions, such as the SCD1 inhibitor aramchol in combination with stearate-supplemented high-fat diet (S-HFD), reduce distant metastasis of CM, presenting a potential strategy for cancer management.

## Methods

### Sex as a biological variable

This study included both male and female human samples, with similar findings observed in both sexes. For animal models, the investigation specifically focused on female mice. It remains unclear whether these findings extend to male mice.

### Specimen Preparation and data collection

The study included CM specimens procured from 36 volunteer patients who were admitted to our hospital between October 2016 and December 2021. These patients underwent resection of clinically and pathologically confirmed CM tissues. The inclusion criteria were as follows: (1) clinical imaging and ophthalmological examination indicating a high likelihood of choroidal melanoma; (2) absence of other pathologies; (3) patients having undergone enucleation surgery at our hospital, with CM diagnosed through intraoperative frozen images and postoperative pathological findings; (4) no evidence of optic nerve invasion by the tumors, as confirmed by intraoperative pathology; (5) postoperative examination of optic nerve tissue showing no presence of tumor cells; (6) all tumors being confined to the intraocular region. Normal choroidal tissues were obtained from mechanically injured or atrophying eyes. Each participant provided written authorization, and the ethics board of The First Hospital of China Medical University granted protocol approval. The collected specimens were split into two segments: one preserved in formalin solution, while the other remained stored at −80 °C. Demographic data and comprehensive patient information appear in Additional File 1: Table S1 and Additional File 2.

### Cells and cell culture

The CM cell lines C918 (RRID: CVCL_8471) and MUM-2B (RRID: CVCL_3450) were provided by the Chinese Academy of Sciences in Shanghai, China, while human epidermal melanocyte cell line PIG1 (RRID: CVCL_7207) cells were obtained from Shanghai Binsui Biotechnology Co., Ltd and is used as a control. C918 cells were cultured in RPMI 1640 medium (Gibco, Thermo Fisher Scientific), MUM-2B cells were cultured in Dulbecco’s modified Eagle’s medium (DMEM, Invitrogen), and PIG1 cells were cultured in Ham’s F12K medium (Gibco). All cell lines were cultured with 10% fetal bovine serum (PAA Laboratories, Pasching, Austria) at 37℃ in a 5% CO₂ atmosphere. All cell lines were routinely tested for mycoplasma and confirmed to be negative.

### m^6^A-dot blot assays

Total RNA extraction was executed utilizing TRIzol reagent (15596018, Invitrogen) per the supplier’s protocol. A total of 1500 ng of RNA was extracted from each sample. After extraction, RNA-free water was added to achieve a final volume of 24 µl, succeeded by the addition of 72 µl of 3 × RI Buffer. The mixture was subjected to incubation at 65 °C for 5 min, allowed to cool to ambient temperature, before introducing 96 µl of 20 × SSC. The sample was loaded into the sampling machine, and each well was rinsed with 10 µl of 10 × SSC. The film was then removed, allowed to dry, and subjected to purple diplomatic couching for 5 min on both sides, followed by methylene blue staining (HY-14536, MCE) for another 5 min. After cleaning with deionized water to ensure clear visibility of the sample points, the sample was scanned for sample control. A 5% milk powder sealing film was applied and left for 1–2 h, followed by washing with PBST twice for 1 h each time. The membrane was incubated overnight at 4 °C with m^6^A antibody (68055-1-lg, Proteintech) at a dilution of 1:2000. The following day, the membrane received three PBST washes lasting 30 min per wash and proceeded to incubate with rabbit secondary antibody for 2 h. Following this incubation period, the membrane underwent three additional PBST washes, each spanning 30 min. Visualization of the membrane was performed using the Fluor Chem V2.0 imaging system.

### Western blotting

The extraction of total protein from tissue or cell lysates utilized a buffer comprising 1% phenylmethylsulfonyl fluoride (PMSF). A bicinchoninic acid (BCA) assay kit (Beyotime Institute of Biotechnology) enabled protein concentration measurement. Following SDS-PAGE separation, proteins underwent transfer onto a polyvinyl formal (PVF) membrane, which received blocking treatment with 5% fat-free milk. The membrane remained sealed within a container during incubation at 37 °C for 60 min. Subsequently, the membranes underwent overnight incubation at 4 °C alongside specific primary antibodies: anti-METTL14 (51104, 1:1000, CST), anti-SCD1 (sc-58420, 1:500, Santa Cruz), anti-β-actin (3700 S, 1:1000, CST), and anti-MAFG (PA5-30086, 1:1000, Invitrogen). The protocol employed peroxidase-conjugated secondary antibodies: goat anti-mouse and goat anti-rabbit IgGs (Abcam). Following triple TBST washing, the membranes underwent incubation with appropriate secondary antibodies at 37 °C for 1 h. Signal detection occurred through an EasySee Western Blot kit (Beijing Transgen Biotech) and chemiluminescence system (Bio-Rad). The intensities of the bands were quantified using ImageJ software (version 1.53a).

### Lentivirus transfection

Plasmids for knocking down and overexpressing METTL14, SCD1, and MAFG were obtained from GeneChem in Shanghai, China. Lentivirus transfections were performed per the supplier’s protocols. Stable transformants were selected over a period of two weeks utilizing puromycin (5 µg/ml). Cells with confirmed knockdown and overexpression of METTL14, SCD1, and MAFG were prepared for subsequent experiments. The transcript numbers for the overexpressed constructs were as follows: METTL14 (NM_020961.4), SCD1 (NM_005063.5), and MAFG (NM_002359.4). The nucleotide sequences of the shRNAs targeting these genes are provided in Additional File 1: Table S2-S4.

### Multiplexed immunohistochemistry (mIHC)

For the mIHC assay, 4 μm formalin-fixed, paraffin-embedded tissue sections were stained using standard primary antibodies along with the TSA 7-color kit (abs50015-100T, Absinbio). Following deparaffinization, sections were incubated with the anti-IL-10RA antibody (#ab225820, Abcam) for 30 min, then treated with a secondary antibody (abs50015-02, Absinbio) targeting rabbit/mouse horseradish peroxidase (HRP)-conjugated for 10 min. TSA 520 was applied for 10 min to develop the sections. After rinsing with TBST buffer, the slides were immersed in preheated citrate solution (90 °C) and microwaved at 20% maximum power for 15 min. Slides were washed with Tris-buffer between each step and allowed to cool to room temperature in the same buffer. DAPI (abs47047616, Absinbio) was applied to the slides, followed by a wash in distilled water and manual cover slipping. Image analysis was performed using Indica Halo software after the slides were air-dried and images were captured using the Pannoramic MIDI II (3DHISTECH). DAPI staining served as a reference for counting cells within the same tissue section, while cells expressing the three target proteins (METTL14, SCD1, and MAFG) were counted as positive cells. The percentage of these positive cells was then computed and subjected to statistical analysis. The antibodies used in the experiment included METTL14 (48699 S, diluted at 1:200, CST), SCD1 (2794 S, diluted at 1:200, CST) and MAFG (NBP2-15019, diluted at 1:200, Novus).

### Lipidomics profiling in CM samples

CM cells were engineered with stable knockdowns of METTL14 and SCD1, as well as control cells, for subsequent lipidomic analysis. Lipidomics sequencing was performed by Novogene Co. Ltd. (Beijing) using LC-MS technology. The process included sample collection, lipid extraction, LC-MS/MS signal detection, and data analysis. Mass spectrometry data underwent processing utilizing Compound Discoverer 3.1 to obtain qualitative and quantitative lipid compound results through spectrogram processing and database searches. Quality control was applied to ensure precision and reliability of the data. Differential metabolic patterns among different groups were analyzed using multivariate statistical methods, including principal component analysis (PCA) and partial least squares discriminant analysis (PLS-DA). Additionally, hierarchical clustering (HCA) and correlation analyses were performed to identify the interrelations between samples and lipid compounds.

### Immunohistochemical staining

The specimens were preserved in 4% paraformaldehyde, processed into paraffin blocks, and cut to obtain 4-µm sections. The prepared slices underwent deparaffinization in xylene, sequential ethanol gradient dehydration, and heat-mediated antigen recovery in citrate buffer using a pressure cooker. Subsequently, the samples were maintained at 4 °C overnight with primary antibodies against METTL14 (dilution 1:1000, HPA038002, Sigma-Aldrich), SCD1 (dilution 1:500, 2794 S, CST), and MAFG (dilution 1:500, #ab154318, Abcam). Microscopic visualization was performed using an inverted microscope (EVOS XL system, AMEX1200; Life Technologies Corp).

### Criteria for immunohistochemical results

The criteria for assessing the expression of immunohistochemistry (IHC) markers (MAFG, METTL14, and SCD1) were defined based on three expression levels observed in tissue samples. Both the nucleus and cytoplasm were examined under the microscope, where blue staining or lack of color indicated negative expression (Grade 1), brownish yellow or light-yellow staining indicated moderate positivity (Grade 2), and dark brown or brown staining indicated strong positivity (Grade 3). Ten high-power fields were randomly selected from each tissue slice, and approximately 100 cells showing positive expression were counted at high magnification. The frequency and extent of positive expression were recorded. Total expression was calculated by multiplying the expression grade by the mean expression area score, yielding values ranging from 0 to 300%.

### Colony formation assay (CFA)

For the colony formation assay, 2 ml of medium containing 1000 cells was seeded into a 6-well plate, and the medium was refreshed every 3 to 4 days. After 7–14 days, colonies were stained with 0.25% crystal violet. Cloning efficiency was determined utilizing ImageJ software (version 1.53a).

### Free fatty acids assay

The Non-esterified Free Fatty Acids (NEFA/FFA) Colorimetric Assay Kit (Elabscience, Catalog No. E-BC-K013-M) was used for free fatty acid analysis, and all procedures followed the manufacturer’s protocol. The protein concentration in the cells was standardized to 10 µl. In the blank well, 10 µl of double-distilled water was added; in the standard well, 10 µl of a 1 mmol/L standard solution was added; and in the sample well, 10 µl of the sample was added. Following this, 200 µl of enzyme reagent was added to each well. The optical density (OD) value A1 of each well was measured at 546 nm using a plate shaker for 10 s at 37 °C over 5 min. Afterward, 50 µl of color developer was added to the wells, and the reaction was allowed to proceed at 37 °C for 5 min. The OD values A2 and ΔA (calculated as A2 - A1) were then determined at 546 nm. OD values were detected using the Infinite F50 (Tecan).

### Reactive oxygen species (ROS) detection (DCFH-DA probe method)

Reagent 1 working solution was prepared by diluting the reagent with serum-free cell culture medium to a final concentration of 10 µM, as validated in preliminary experiments. The reagent was diluted at a 1:1000 ratio to exclude potential interference from DMSO. For application, cells were suspended in 2 ml Eppendorf tubes, and 500 µl of the working solution was added. The tubes were incubated at 37 °C for 30 min, with gentle shaking every 10 min. In cases where a pronounced ROS effect was expected, the working solution was introduced following pre-treatment with the reagent. Due to the increased ROS generation induced by Reagent 2 (ROS up), a significant difference was observed between the treated and control groups using the same cell type. Notably, ROS up was designated for positive controls, with no additional treatments applied to other groups. Reagent 2 was used solely to assess the compatibility of the CFA-DH probe within this experimental framework. Flow cytometry analysis, including FITC-A channel detection, was performed using FlowJo V10 software. Measurements were acquired with a BD FACSCelesta (BD Biosciences).

### Membrane fluidity analysis

The Membrane Fluidity Kit (ab189819, Abcam) was utilized according to the manufacturer’s protocol. Cell membrane fluidity changes were assessed using both microscopic analysis. For microscopic analysis, adherent cells were seeded in suspension in a multi-well plate. The preparation included a labeling mixture containing 5 µM Fluorescent Lipid Reagent combined with Perfusion Buffer (designated for adherent cells). Subsequently, the cellular components underwent incubation with the prepared reagent at 25 °C for 1 h in dark conditions. After incubation, unincorporated PDA was removed by washing the cells twice with perfusion buffer. The cells were then resuspended in a medium. Monomer and excimer fluorescence were measured using a Fluorescence Microplate Reader, with fluorescence detection at 400 nm and 470 nm, utilizing a 350 nm excitation filter.

### Mouse tail vein injections induce hepatopulmonary metastasis

Female BALB/c nude mice, aged 6 weeks, were used in this study. The mice were procured from Beijing Vital River Experimental Animal Technology Co., Ltd. and maintained in the Experimental Animal Department of China Medical University. After treatment, roughly 1 × 10^5^ cells were administered to each mouse group through gradual injection methods with a 100 µl volume. The mice were divided into experimental groups to investigate the effects of dietary conditions and therapeutic interventions on tumor metastasis. Prior to injection, the mice were acclimatized to a chow diet for three days before being assigned to the experimental groups. The groups were fed either a normal-fat diet (NFD; 12.0% fat; F-2, Oriental Yeast, Tokyo), a high-fat diet enriched with stearate (S-HFD; 60% fat; D12113001, Research Diets, New Brunswick, NJ), or a high-fat diet enriched with oleic acid (O-HFD; 56.7% fat; HFD32, CLEA Japan, Tokyo), with these diets maintained throughout the study. After a 1.5-month observation period, whole-body PET scans were executed. The lungs and liver were harvested for further analysis. Histological sections were stained with HE for analysis. The research protocol (CMU2021090) was sanctioned by the Ethics Committee of the First Hospital of China Medical University.

### Characterization by PET imaging

PET data underwent processing utilizing Metis animal PET system software (MadicPet V1.0.4). Mice received an intravenous injection of approximately 100 µCi of [^18^F] fluoro-D-glucose. After anesthesia with isoflurane, the mice were placed in a prone position in the scanner. The movement of the scanning bed was accurate to 0.05 mm. PET metadata were generated, and images were reconstructed and analyzed using Metis animal PET system software.

### Hematoxylin and Eosin (H&E) staining of paraffin-fixed tissues

Paraffin-embedded lung and liver tissue sections from nude mice were deparaffinized with xylene. The sections were then rehydrated through a graded series of ethanol solutions (100%, 96%, and 75% ethanol). After rehydration, the sections were stained with hematoxylin for 2 min, differentiated in tap water for 15 min, and then incubated with eosin for 1 min. Following staining, the sections were dehydrated through increasing concentrations of ethanol and then cleared in xylene for 1 min.

### Oil red O (ORO) staining

ORO staining was performed according to the manufacturer’s protocol (C0157M, Beyotime). Sections were initially treated with a suitable volume of dye wash solution for 20 s. After discarding the wash solution, the prepared ORO staining solution was added and incubated for 20 min. The staining solution was then removed, followed by a 30-second rinse with the dye wash solution. Sections were subsequently immersed in distilled water and gently agitated for 20 s. Finally, sections were sealed using glycerin gelatin sealing solution (C0187, Beyotime). Stained sections were visualized and imaged using an inverted microscope (OLYMPUS).

### RNA extraction and transcriptome sequencing

Transcriptomic analysis of CM cells with METTL14 knockdown or overexpression was outsourced to Gene Denovo Corporation (Guangzhou) for sequencing. Total RNA was extracted using the Trizol reagent kit (Invitrogen) according to the manufacturer’s instructions. RNA quality was evaluated with an Agilent 2100 Bioanalyzer (Agilent Technologies) and confirmed through agarose gel electrophoresis. mRNA enrichment was performed prior to sequencing. The cDNA library was sequenced by Gene Denovo Biotechnology Co. (Guangzhou) using the Illumina Novaseq 6000 platform.

### RNA extraction and qRT-PCR assay

cDNA synthesis was performed utilizing PrimeScript™ RT Master Mix (Takara Biotechnology), while RT-PCR amplification occurred through SYBR Premix Ex Taq™ (Takara Biotechnology). RNA examination took place on a Light Cycler 480 II system (Roche), employing β-actin as the internal reference. mRNA expression levels underwent quantification via the 2^−ΔΔCt^ method. The specific primer sequences applied for reverse transcription polymerase chain reaction (RT-PCR) and quantitative polymerase chain reaction (qPCR) amplification are listed in Additional file 1: Table S5.

### Fluorescence in situ hybridization (FISH) assay

Experimental cells under varying treatments were distributed in 6-well plates with 6 × 10^4^ cells introduced to each well. The supernatant portion was extracted, and the cells underwent fixation using paraformaldehyde at 4°C. Post-prehybridization, the samples received probes and remained at 40 °C during a 22–24 h period. Three sequential washes with sodium citrate were performed on the sections, followed by a 15-minute exposure to hybridization solution. Subsequently, the sections underwent PBS rinsing, enabling result collection. FISH probe sequences are available in Additional file 1: Table S6.

### M^6^A RNA Immunoprecipitation (MeRIP) assay

The MeRIP analysis was performed utilizing an N6-Methylated RNA Immunoprecipitation (MeRIP) Kit (Bes5203, BersinBio) per the supplier’s protocols. Initially, RNA extraction and chemical fragmentation were carried out. Subsequently, the fragmented RNA underwent incubation with either N6-methyladenosine antibody (ab208577, Abcam) or control immunoglobulin G (IgG) antibody. Magnetic bead-based separation was implemented for isolation. Following RNA enrichment through phenol-chloroform extraction combined with ethanol precipitation, quantitative PCR assessed the influence of m^6^A modifications on specific genes. The m^6^A locations of particular genes were obtained from the SRAMP database (http://www.cuilab.cn/sramp). MeRIP-qPCR primer sequences are listed in Additional File 1: Table S7.

### Dual luciferase reporter assay

The luciferase plasmid was transfected into individual wells of a 24-well plate using Lipofectamine™ 3000 (Invitrogen). Firefly and Renilla luciferase activities were measured 48 h post-transfection using the Dual-Luciferase Assay kit (Promega). Relative luciferase activity was quantified with a Synergy HTX multimode microplate reader (BioTek).

### Transwell assay

Matrigel-coated (BD) 8-µm-pore transwell chambers (Corning) were used. Transfected cells (1 × 10^6^) were added to the upper chamber in 200 µl of the serum-free growth medium, while 600 µl growth medium containing serum was placed in the lower chamber. Following 24-hour cultivation, the cells underwent fixation using 4% paraformaldehyde for 20 min, followed by crystal violet staining and PBS washing before quantification. An inverted microscope captured the images, and cell enumeration was conducted utilizing ImageJ (1.53a) software.

### Statistics

Data analysis was conducted using GraphPad Prism 8.0 (GraphPad) and Microsoft Office 2019 Excel. The correlation between variables was assessed utilizing determination coefficient analysis. Statistical significance was assessed using Student’s t-test, with a threshold of *P* < 0.05. Detailed statistical tests for each experiment are provided in the figure legends, where *P* < 0.05 indicates significance and “ns” denotes no significant difference.

## Results

### METTL14 enhances the invasion and metastasis of CM by mediating lipid metabolism pathways to facilitate membrane fluidity

**Fig. 1:**
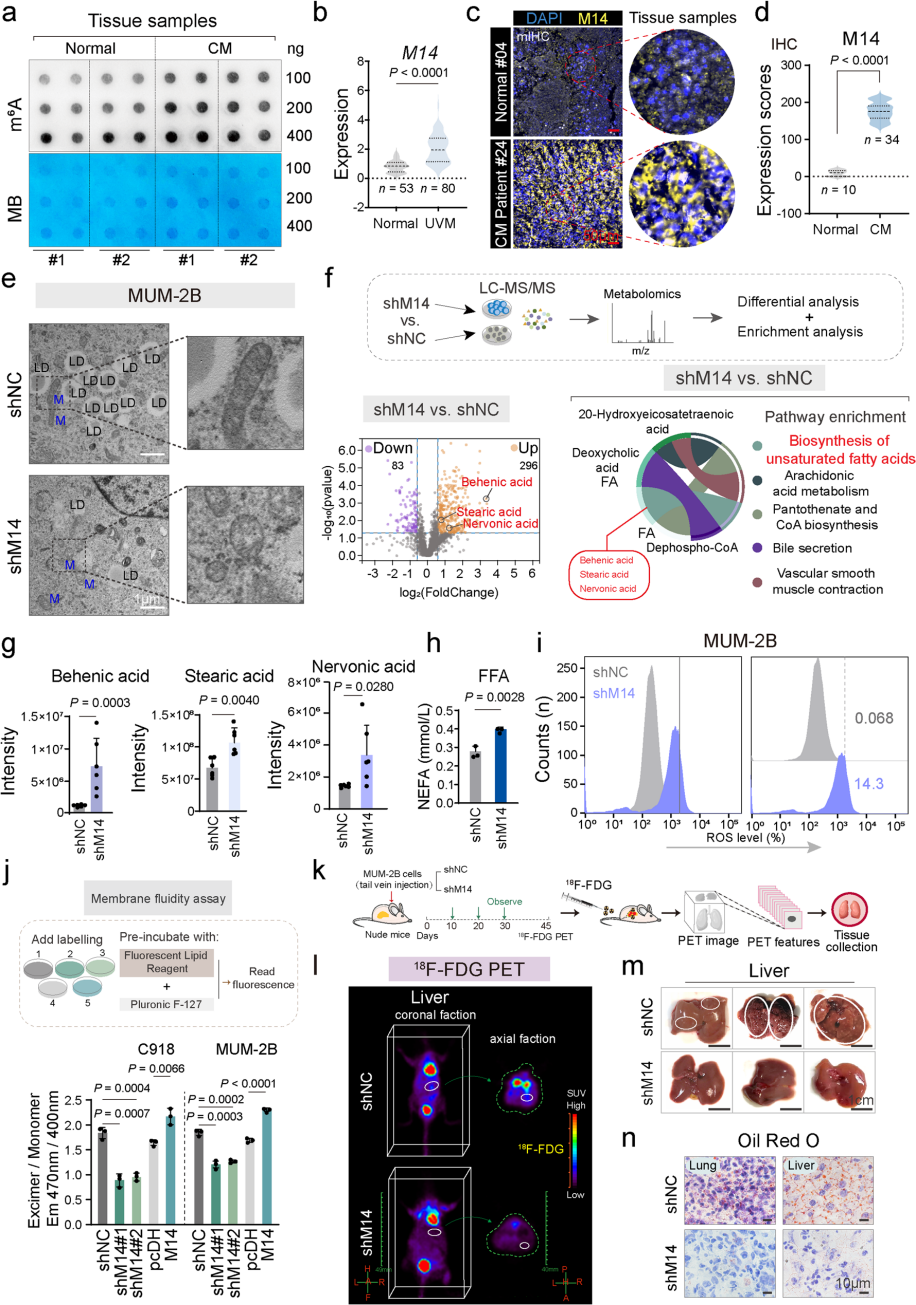
METTL14 promotes choroidal melanoma invasion and metastasis by mediating lipid metabolic pathways to facilitate membrane fluidity. **a**, m^6^A dot blot assays demonstrating the upregulation of m^6^A modification levels in tissues of patients with CM. **b**, Differential expression of METTL14 in normal individuals and patients with UVM, as represented by the GTEx and TCGA databases. (GTEx: Normal, *n* = 53; TCGA: UVM, *n* = 80). **c**, Representative multiplexed immunohistochemistry staining images of normal choroidal samples and CM samples. METTL14 (yellow), nuclei stained with DAPI (blue). Circular regions are shown at higher magnification on the right. **d**, IHC assay detecting METTL14 protein expression in normal choroid tissues (Normal: *n* = 10) and CM tissues (CM: *n* = 34). **e**, Transmission electron microscopy (TEM) analysis showing lipid droplet reduction and mitochondrial contraction after silencing METTL14 in MUM-2B cells. LD, lipid droplet; M, mitochondria. Scale bars: 1 μm. **f**, Non-targeted metabolomics analysis of shNC and METTL14 knockdown MUM-2B stable cells. Volcano plot showing 83 downregulated and 296 upregulated differential metabolites in METTL14-treated CM cells (MUM-2B) (FC > 1.5, *P* < 0.05) compared to the control group. KEGG enrichment analysis of metabolic pathways related to the differential metabolites. **g**, Peak area values of Behenic acid, Stearic acid, and Nervonic acid in the unsaturated fatty acid biosynthesis pathway, comparing the shNC and shM14 groups. **h**, Free fatty acid colorimetry (NEFA/FFA) revealing increased free fatty acid content after silencing METTL14. **i**, Flow cytometry assays detecting ROS levels after interfering with METTL14 expression in CM cells. **j**, Membrane fluidity test showing changes in cell membrane fluidity after interfering with METTL14 expression in C918 and MUM-2B cells. **k-l**, Representative coronal and axial sectional PET images showing the uptake of ^18^F-FDG in the liver of mice in two different groups (shNC and shM14, *n* = 5 for each). **m**, Representative images of livers excised from mice injected with METTL14-silencing and negative control cells (scale bar: 1 cm). **n**, Representative Oil Red O (ORO) staining images of mouse lung and liver tissues in two different groups (shNC and shM14, *n* = 5 for each)

To explore the epigenetic regulatory role of RNA m^6^A methylation in CM, m^6^A methylation abundance was evaluated between CM and normal choroidal tissues using the RNA m^6^A dot blot assay. Clinical case analysis demonstrated a notable elevation of m^6^A expression in CM patient tissues (*P* < 0.05, Fig. 1a). Moreover, analysis of METTL14 mRNA expression in 10 tissues of patients with CM compared to 10 normal choroidal tissues, we identified METTL14 as the significantly upregulated gene in CM tissues relative to normal choroidal tissues (Figure S1a). Subsequently, differential expression of *RNA* m^6^A writer protein, METTL14, was analyzed using data from the GTEx (Normal: *n* = 53) and TCGA (UVM: *n* = 80) platforms. The analysis revealed a notable upregulation of METTL14 in UVM (*n* = 80) compared to normal tissues (*n* = 53) (Fig. 1b). Investigation of METTL14 protein expression in choroidal melanoma cells demonstrated a significant increase in C918 and MUM-2B cells (*P* < 0.05, Figure S1b), supporting the association between elevated m^6^A expression and METTL14 upregulation in uveal melanoma.

mIHC assays of clinical specimens further validated these observations, confirming a notable elevate in METTL14 level in CM tissues (Fig. 1c). Moreover, analysis of METTL14 expression in 34 tissues of patients with CM compared to 10 normal choroidal tissues revealed abnormal overexpression of METTL14 in CM tissues (Fig. 1d), indicating aberrant METTL14 protein overexpression in both CM cells and tissues. Additionally, WB analysis revealed that METTL14 protein levels were downregulated as a result of transfection (*P* < 0.05, Figure S1c). Previous studies demonstrated that METTL14 promotes CM invasiveness, and additional evidence suggests its role in CM proliferation. Specifically, CFA experiments showed that silencing METTL14 in C918 cells impaired their clonogenic ability, whereas overexpression of METTL14 enhanced clonogenic formation (Figure S1d). To assess METTL14 inhibition-induced ultrastructural changes in CM cells, transmission electron microscopy (TEM) was performed. Furthermore, TEM assays revealed that silencing METTL14 markedly reduced lipid droplet formation and induced mitochondrial contraction in MUM-2B cells (Fig. 1e). These results suggest a close relationship between METTL14, lipid droplet formation, and lipid metabolism, though the underlying molecular mechanisms remain to be fully elucidated.

To fully elucidate the impact of METTL14 on cellular metabolism in CM cells, lipidomics analysis was conducted following METTL14 silencing in MUM-2B cells (Fig. 1f). The analysis revealed 2964 distinct metabolites, comprising 1679 detected in positive ion mode and 1285 in negative ion mode, along with the identification of 22 specific lipid categories. Silencing METTL14 led to the downregulation of 83 lipid metabolites and the upregulation of 296 lipid metabolites (FC >1.5, *P* < 0.05). Enrichment analysis of differentially expressed metabolites indicated notable concentration in the unsaturated fatty acid synthesis pathway. Notably, after METTL14 suppression, the peak areas of Behenic acid, Stearic acid, and Nervonic acid in this pathway showed substantial increases (Fig. 1g). Further analysis demonstrated a marked elevation in the concentration of free fatty acids within the cells following METTL14 inhibition (Fig. 1h). Free fatty acids have been demonstrated to trigger ROS generation [37], and indeed, ROS levels markedly increased after METTL14 silencing (Fig. 1i).

To explore the consequences of METTL14 inhibition further, the accumulation of free fatty acids and ROS in CM cells was evaluated. The findings suggested that METTL14 suppression markedly enhanced the accumulation of free fatty acids and ROS production compared to control-treated cells. Consistent with the results in Fig. 1e, the elevated ROS levels contributed to mitochondrial structural disruption., mitochondria in METTL14-silenced cells became fragmented, rounded, and lost their cristae. Previous studies have suggested that Behenic acid and Stearic acid can be incorporated into the fatty acid chains of glycerophospholipids, promoting closer interactions between these molecules, which in turn reduces membrane fluidity [38, 39]. Membrane fluidity regulation is critical for tumor invasion and metastasis [6, 40], and our analysis revealed that silencing METTL14 decreased membrane fluidity, while METTL14 overexpression increased membrane fluidity in CM cells (Fig. 1j). These results underscore the pivotal role of METTL14 in maintaining cell membrane dynamics. ^18^F-FDG PET/CT is a reliable imaging modality for characterizing malignancy grade and metastatic burden while enabling visualization of metabolic activity in viable tumor cells and has been widely used in clinical cancer diagnosis and management [41, 42]. MUM-2B cells were stably transfected with shNC and shMETTL14, and a nude mouse CM metastasis model was established *via* tail vein injection of 1 × 10^5^ cells. After 45 days, in vivo PET imaging with ^18^F-FDG was executed to assess the impact of METTL14 silencing on CM metastasis and metabolism in nude mice (Fig. 1k). The findings exhibited a notable reduction uptake of ^18^F-FDG in liver and lung metastases [30] in the shMETTL14 group versus the shNC group (Fig. 1l and Figure S1e). Gross anatomy analysis revealed that the shNC group mice exhibited liver metastasis, whereas no macroscopic liver metastases were observed in the METTL14-silenced group (Fig. 1m and Figure S1f). Histological examination using H&E staining confirmed the absence of overt liver metastatic lesions in the METTL14-silenced mice (Figure S1g). IHC analysis suggested a marked decline in METTL14 expression levels in the organs of METTL14-silenced nude mice (Figure S1h). Additionally, ORO staining demonstrated a marked diminish in lipid deposition in the organs of these mice (Fig. 1n and Figure S1i). Collectively, these results suggest that METTL14 facilitates CM invasion and metastasis through lipid metabolism, while its silencing impairs membrane fluidity, a process mediated by key components such as Behenic acid and Stearic acid.

### METTL14 regulates lipid metabolism component alterations mediated by SCD1

**Fig. 2:**
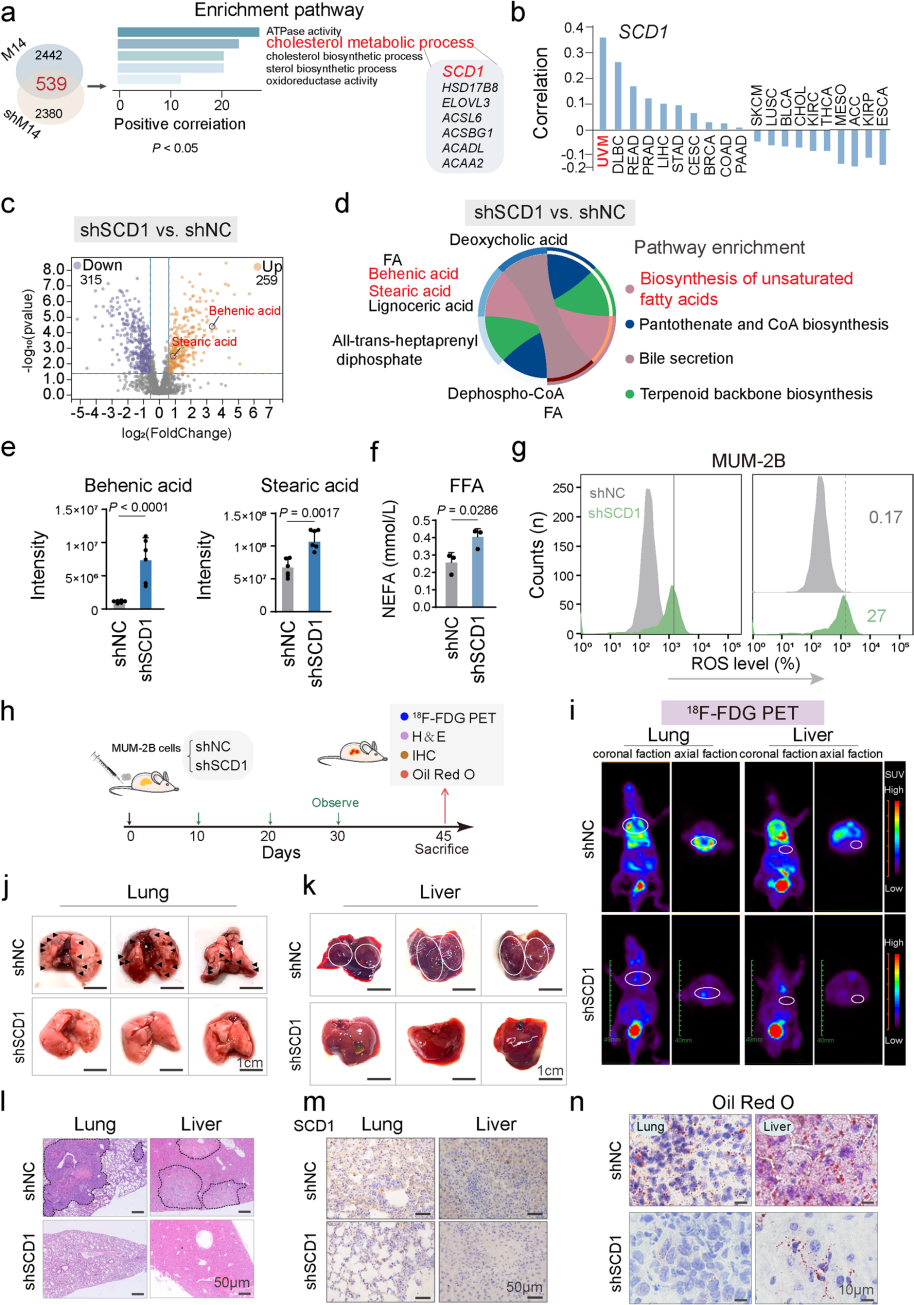
METTL14 mediates lipid metabolism component alterations through SCD1 to promote the characteristics of choroidal melanoma metastasis.** a**, Venn diagram and pathway enrichment analysis identifying *SCD1*,* HSD17B8*,* ELOVL3*,* ACSL6*,* ACSBG1*,* ACADL*, and *ACAA2* as downstream target candidates of METTL14. **b**, RM2target (http://rm2target.canceromics.org/) showing the correlation between SCD1 and METTL14 in various cancers. **c**, Volcano plot showing 315 downregulated and 259 upregulated differential metabolites in SCD1-treated CM cells (MUM-2B) (FC > 1.5, *P* < 0.05) compared to the control group. **d**, KEGG enrichment analysis of metabolism pathways associated with differential metabolites. **e**, Peak area values of Behenic acid and Stearic acid in the unsaturated fatty acid biosynthesis pathway, comparing the shNC group vs. shSCD1 group. **f**, Free fatty acid colorimetry (NEFA/FFA) showing an increase in free fatty acid content after silencing SCD1. **g**, Flow cytometry showing ROS levels after silencing SCD1. **h-i**, Representative coronal and axial sectional PET images showing the uptake of ^18^F-FDG in the lung and liver of mice in two different groups (shNC and shSCD1, *n* = 5 for each). **j-k**, Representative images of excised lungs and livers from mice injected with the indicated cells. (Scale bar, 1 cm). **l**, Representative hematoxylin and eosin (H&E) staining images of lung and liver tissues. **m**, Immunohistochemical analysis of mouse lung and liver tissues with an anti-SCD1 antibody in two different groups (shNC and shSCD1, *n* = 5 for each). Scale bar, 50 μm. **n**, Representative ORO staining images of frozen mouse lung and liver tissues

To further elucidate how METTL14 regulates abnormal lipid metabolism in CM by triggering its downstream key pathways, enrichment analysis was conducted using RNA-seq datasets from METTL14 silencing and overexpression experiments. The analysis identified 539 downstream target genes positively correlated with METTL14 expression, which were defined as genes that were upregulated upon METTL14 overexpression (*P* < 0.05) and downregulated upon METTL14 knockdown (*P* < 0.05). KEGG enrichment analysis of these genes revealed the top five most significant signaling pathways: ATP enzyme activity, cholesterol metabolism, cholesterol biosynthesis, steroid biosynthesis, and oxidoreductase activity (Fig. 2a). Given that METTL14 contributes to oncogenesis by facilitating CM invasion and metastasis through lipid metabolism, lipid metabolism-related pathways were examined. By analyzing the correlation between SCD1 and METTL14 in various tumors and assessing the association of these genes with UVM prognosis using TCGA data, it was concluded that SCD1 is a potential key target gene regulated by METTL14 and mediates poor prognosis in UVM (Fig. 2b and Figure S1a).

To gain a comprehensive understanding of how SCD1 inhibition disrupts CM lipid homeostasis, SCD1 was silenced in MUM-2B cells, followed by lipidomic analysis using LC-MS/MS. The analysis demonstrated that SCD1 knockdown resulted in decreased levels of 315 lipid metabolites and elevated levels of 259 lipid metabolites (FC > 1.5, *P* < 0.05, Fig. 2c). KEGG enrichment analysis of the differential metabolites showed results consistent with those observed after METTL14 silencing, with the unsaturated fatty acid synthesis pathway showing the most significant enrichment (Fig. 2d). Notably, the peak areas of Behenic acid and Stearic acid in this pathway markedly increased following SCD1 silencing, mirroring the trend observed after METTL14 silencing (Fig. 2e). Silencing SCD1 in CM cells resulted in a significant accumulation of free fatty acids and elevated ROS levels (Fig. 2f, g).

To assess the impact of SCD1 on CM metastasis in vivo, two stable MUM-2B cell lines (1 × 10^5^ cells) transfected with shNC or shSCD1 were injected *via* the tail vein to develop a nude mouse CM metastasis model (Fig. 2h). After 45 days, PET imaging revealed markedly diminished uptake of ^18^F-FDG on lung and liver metastases in the shSCD1 group versus the shNC group (Fig. 2i and Figure S1b). Gross anatomical dissection showed extensive lung and liver metastasis in the shNC group, while no macroscopic lung and liver metastases were detected in the shSCD1 group (Fig. 2j, k and Figure S1c). Histological analysis through HE staining confirmed the absence of overt lung and liver metastases in the shSCD1 group (Fig. 2l). IHC assays confirmed a marked decrease in SCD1 level in the organs of the shSCD1 group (Fig. 2m and Figure S1d). ORO staining indicated markedly diminished in lipid deposition in the organs of these mice (Fig. 2n and Figure S1e). In summary, these results demonstrate that SCD1 silencing inhibits CM progression by inducing metabolic stress and reducing metastatic dissemination, accompanied by a decrease in ^18^F-FDG uptake.

### METTL14 activates SCD1 m^6^A methylation to modulate CM lipid metabolism

**Fig. 3:**
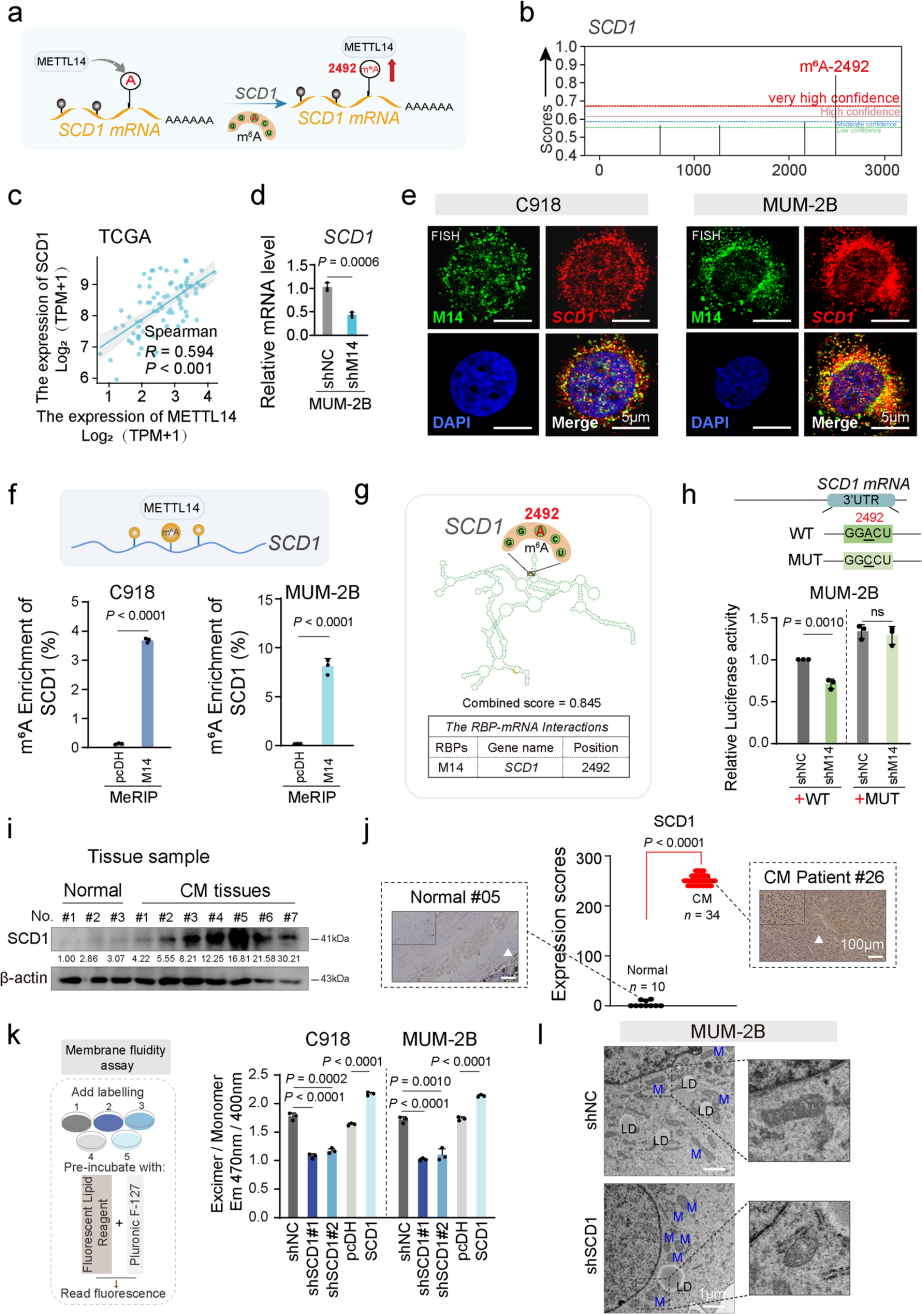
METTL14 mediates choroidal melanoma lipid metabolism by inducing SCD1 m^6^A methylation modification. **a**, Schematic diagram illustrating the METTL14-SCD1 regulatory axis *via* a specific m^6^A modification-dependent mechanism. **b**, The SRAMP (http://www.cuilab.cn/sramp) predicting the distribution of high-confidence m^6^A methylation sites and scores evaluation for SCD1 mRNA. **c**, Linear correlation between METTL14 expression and SCD1 expression. **d**, qRT-PCR analysis showing alterations in SCD1 mRNA levels after METTL14 depletion. **e**, FISH assay showing the expression and co-localization of METTL14 (green) with SCD1 (red) in CM cells. DAPI (blue) is used as the nuclear marker. Scale bar, 5 μm. **f**, m^6^A-MeRIP-qPCR assay showing enrichment of *SCD1* by m^6^A-specific antibodies. **g**, ENCORI-CLIP database (http://starbase.sysu.edu.cn/) illustrating the RBP-mRNA interactions between METTL14 and SCD1. **h**, Double luciferase assay confirming METTL14 directly binds to the 2492 site of SCD1 3’UTR, with mutations at this site. **i**, Representative western blot images showing SCD1 expression in normal choroidal tissues and CM tissues. **j**, Immunohistochemical analysis of human normal choroidal and CM tissue samples with the indicated antibodies. Representative images shown. Scale bar, 100 μm. (Normal: *n* = 10, CM: *n* = 34). **k**, Membrane fluidity assay showing changes in cell membrane fluidity after interfering with SCD1 expression in C918 and MUM-2B cells. **l**, Transmission electron microscopy (TEM) showing lipid droplet reduction and mitochondrial contraction following the silencing of SCD1 in MUM-2B cells. LD, lipid droplet; M, mitochondria. Scale bars, 1 μm

The study investigated the precise regulatory mechanism by which METTL14 targets SCD1 expression, as illustrated in Fig. 3a. Epigenetic transcriptional regulation was examined in this context. The m^6^A distribution of target genes involved in the cholesterol pathway related to lipid metabolism, including *SCD1*, *HSD17B8*, *ELOVL3*, *ACSL6*, *ACSBG1*, *ACADL*, and *ACAA2*, was analyzed utilizing the SRAMP platform (https://www.cuilab.cn/sramp) (Fig. 3b and Figure S3a). Correlation analysis demonstrated a notable positive linear relationship between METTL14 and SCD1 level (*R* = 0.594, *P* < 0.001, Fig. 3c). This finding was further supported by qRT-PCR, which demonstrated that silencing METTL14 substantially decreased *SCD1* mRNA expression (Fig. 3d). To confirm the m^6^A-dependent regulation of *SCD1* by METTL14, FISH assays were executed to observe the co-expression of METTL14 and *SCD1* mRNA in both the nucleus and cytoplasm of C918 and MUM-2B cells. The observed co-localization (Fig. 3e) provided a foundation for future functional investigations. Moreover, Me-RIP assays revealed that elevated METTL14 expression resulted in substantially stronger interaction between SCD1 and the m^6^A antibody (Fig. 3f), indicating that METTL14 enhances SCD1 expression *via* m^6^A-mediated epigenetic modifications. Subsequent analysis identified the specific m^6^A site on *SCD1* targeted by METTL14. SRAMP analysis identified the m^6^A-2492 position in the *SCD1* 3’ UTR region with high confidence. Structural prediction of the *SCD1* mRNA by SRAMP (Fig. 3g) highlighted the key GGACU motif (+ 2490 to + 2494) within the *SCD1* 3’ UTR region, recognized by METTL14 through ENCORI. To further validate this, plasmids containing either the wild-type (WT) motif with GGACU at site A-2492 in the *SCD1* 3’ UTR region or a mutant plasmid with a mutated A-2492 site were constructed. Dual luciferase reporter assays revealed that silencing METTL14 markedly reduced *SCD1* mRNA expression in the WT group, while no change was detected in the mutant group, thereby confirming the critical role of the A-2492 site in METTL14’s epigenetic regulation of *SCD1* mRNA (Fig. 3h).

The impact of METTL14 on the regulation of SCD1 in CM invasion and metastasis was further investigated. Analysis of the GTEx dataset for normal individuals (*n* = 53) and the TCGA UVM dataset for patients with uveal melanoma (*n* = 80) revealed elevated levels of *SCD1* in UVM (Figure S3b). Western blot assays validated a substantial elevation of SCD1 level in clinically derived CM tissues (*n* = 7) compared to normal choroid tissues (*n* = 3, Fig. 3i). Additionally, IHC experiments demonstrated marked upregulation of SCD1 in CM (*n* = 34) compared to normal choroid tissue (*n* = 10, Fig. 3j). Clinical validation across CM cases conclusively demonstrates SCD1 upregulation in CM patient tissues. The influence of SCD1 on cell membrane fluidity and invasive capacity was then assessed. Membrane fluidity experiments showed that downregulation of SCD1 in CM cell lines reduced cell membrane fluidity, while upregulation enhanced membrane fluidity (Fig. 3k), indicating that SCD1 serves a pivotal function in membrane dynamics. Transwell assays further revealed that SCD1 knockdown attenuated the invasive potential of C918 and MUM-2B cells, whereas SCD1 overexpression markedly increased the invasiveness of these CM cell lines (Figure S3c). Notably, TEM analysis showed that suppressing SCD1 expression resulted in a marked decrease of lipid droplets and mitochondrial shrinkage in MUM-2B cells, accompanied by mitochondrial structural damage. The mitochondria in these cells appeared fragmented and rounded, with a loss of cristae, compared to the elongated tubular mitochondria seen in control cells (Fig. 3l). These observations suggest a strong association between the METTL14-mediated *SCD1* m^6^A methylation modification pathway and lipid droplet formation, as well as lipid metabolism.

### The METTL14-SCD1 signaling axis drives lipid metabolism to promote CM progression

**Fig. 4:**
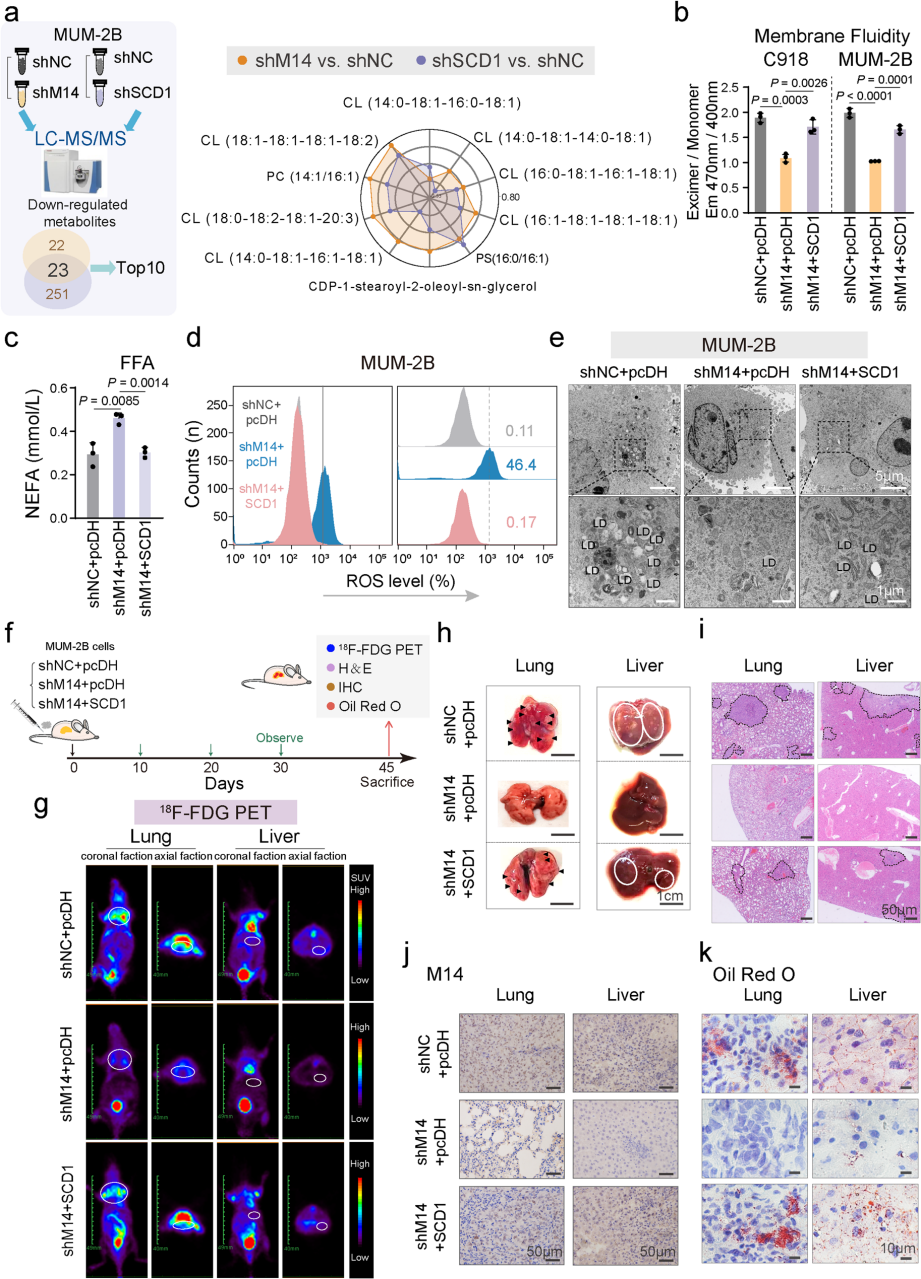
The METTL14-SCD1 signaling axis regulates lipid metabolism to promote CM invasion and metastasis.** a**, Radar chart illustrating the top 10 downregulated differential metabolic products obtained from the shNC + shMETTL14 group vs. shNC + shSCD1 group (*P* < 0.05). **b**, Rescue experiments determining cell membrane fluidity by overexpressing SCD1 upon silencing METTL14 in C918 and MUM-2B cells. **c**, Rescue experiments revealing free fatty acid content treated by overexpression of SCD1 upon silencing METTL14 in MUM-2B cells. **d**, Flow cytometry detecting ROS levels in the recovery experiment groups. **e**, Rescue experiments determining the number of lipid droplets after overexpression of SCD1 upon silencing METTL14 in MUM-2B cells. LD, lipid droplet. Scale bars, 5 μm and 1 μm. **f-g**, Representative coronal and axial sectional PET images showing the uptake of ^18^F-FDG in the lung and liver of mice in three different groups (shNC + pcDH, shM14 + pcDH, and shM14 + SCD1). **h**, Representative images of excised lungs and livers from mice injected with the indicated cells. Scale bar, 1 cm. **i**, Representative images of H&E staining of lung and liver tissues. **j**, Immunohistochemical analysis of mouse lung and liver tissues performed with an anti-METTL14 antibody in three different groups (shNC + pcDH, shM14 + pcDH, and shM14 + SCD1). Scale bars, 50 μm. **k**, Representative ORO staining images of lung and liver tissues

Concurrent inhibition of METTL14 and SCD1 resulted in the downregulation of 23 differential metabolites, with cardiolipin constituting 70% of the top 10 most markedly altered metabolites. Specifically, cardiolipin species such as CL (14:0–18:1–16:0–18:1), CL (14:0–18:1–14:0–18:1), and CL (16:0–18:1–16:1–18:1) were identified as key components in lipid metabolism (Fig. 4a). The roles of METTL14 and SCD1 in regulating CM lipid metabolism and metastasis were further validated both in vitro and in vivo. Rescue experiments determining cell membrane fluidity by overexpressing SCD1 upon silencing METTL14 in C918 and MUM-2B cells (Fig. 4b). Silencing METTL14 decreased cell invasiveness, with partial restoration observed following SCD1 overexpression (Figure S4a). Additionally, METTL14 silencing led to elevated levels of free fatty acids and reactive oxygen species, which were partially reversed by SCD1 overexpression (Fig. 4c, d). TEM analysis further revealed that SCD1 overexpression reversed the reduction in lipid droplets induced by METTL14 silencing (Fig. 4e).

To assess the roles of SCD1 and METTL14 in promoting lung and liver metastasis in vivo, MUM-2B cells (1 × 10^5^ cells per group) were transduced with shNC + pcDH, shMETTL14 + pcDH, and shMETTL14 + SCD1, and a nude mouse CM metastasis model was established *via* tail vein injection (Fig. 4f). Rescue experiments demonstrated that the reduction in lung and liver metastasis caused by METTL14 silencing could be reversed by SCD1 overexpression (Fig. 4g-i and Figure S4b, c). IHC analysis showed a significant decrease in METTL14 expression in lung and liver metastases in the shMETTL14 + pcDH group versus the shNC + pcDH group, with expression levels restored to baseline in the shMETTL14 + SCD1 group (Fig. 4j and Figure S4d). ORO staining demonstrated a notable reduction in lipid deposition in the lung and liver metastases of the shMETTL14 + pcDH group, with restoration upon SCD1 overexpression (Fig. 4k and Figure S4e). These findings suggest that elevated SCD1 expression substantially reverses the reduction in lung and liver metastasis and lipid deposition induced by METTL14 silencing in nude mice. These results highlight METTL14’s role in regulating lipid metabolism and promoting cancer cell invasion and metastasis by directly targeting the mRNA of *SCD1*.

### METTL14 is activated by transcriptional activator MAFG in CM

**Fig. 5:**
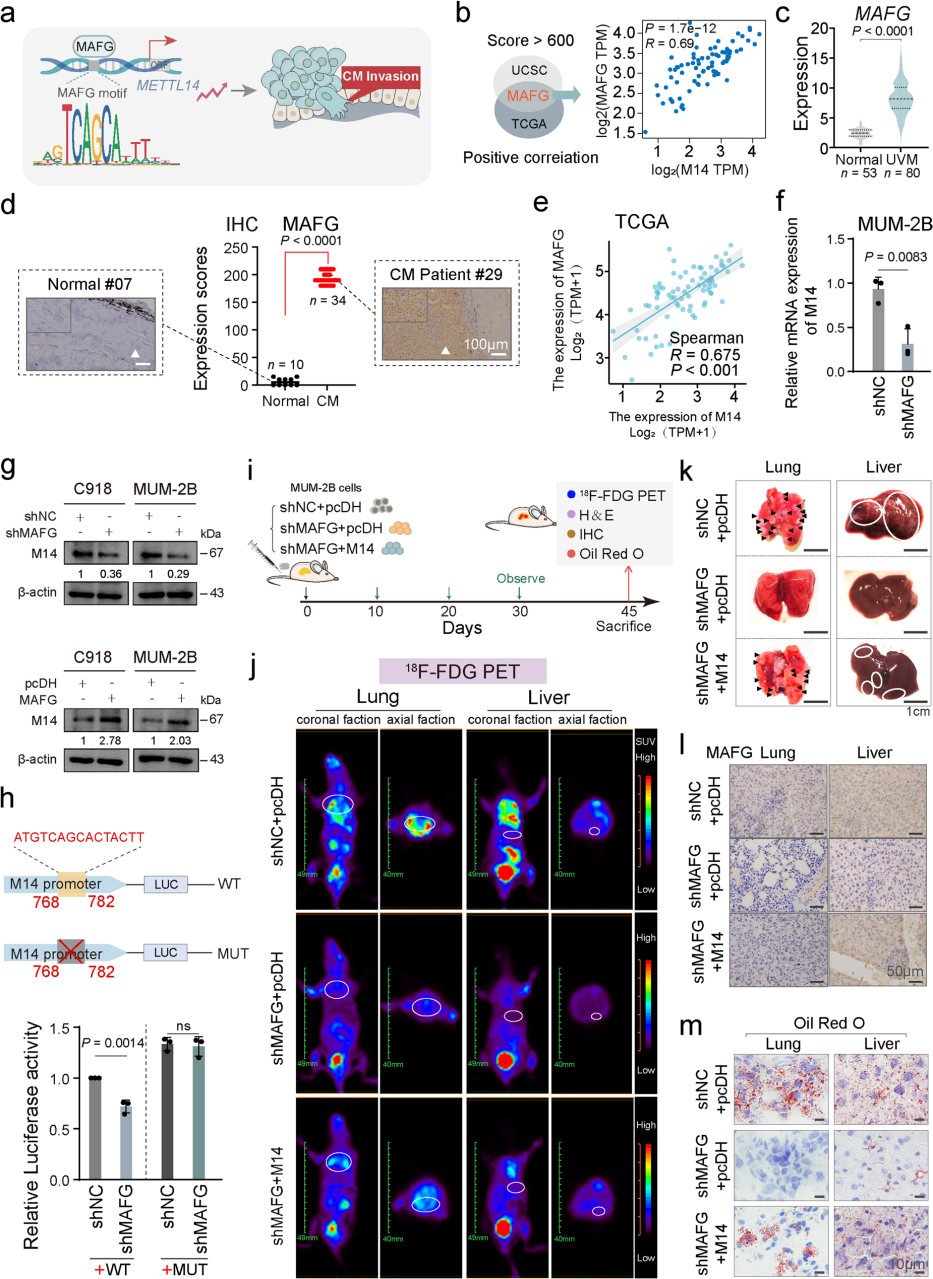
METTL14 in CM is induced by MAFG through the activation of the response element.** a**, Schematic diagram illustrating how MAFG transcriptionally activates METTL14 to regulate CM cell invasion properties. **b**, UCSC (https://xena.ucsc.edu/) and TCGA (https://portal.gdc.cancer.gov/) predicting MAFG as the transcriptional activator of METTL14. **c**, GTEx (https://www.genome.gov/Funded-Programs-Projects/Genotype-Tissue-Expression-Project) and TCGA (https://portal.gdc.cancer.gov/) databases showing high expression of MAFG in patients with UVM (Normal: *n* = 53, UVM: *n* = 80). **d**, Immunohistochemical analysis of human normal choroidal and CM tissue samples with the indicated antibodies. Representative images shown. Scale bar, 100 μm. (Normal: *n* = 10, CM: *n* = 34). **e**, Linear correlation between METTL14 expression and MAFG expression. **f**, qRT-PCR analysis of METTL14 mRNA levels after MAFG inhibition. **g**, Representative western blot images showing METTL14 expression in CM cells after interfering with MAFG expression. **h**, Double luciferase assay confirming specific binding sites of MAFG to the METTL14 promoter after mutations at the METTL14 promoter. **i-j**, Representative coronal and axial sectional PET images showing the uptake of ^18^F-FDG in the lung and liver of mice in three different groups (shNC + pcDH, shMAFG + pcDH, and shMAFG + M14). **k**, Representative images of excised lungs and livers from mice injected with the indicated cells. Scale bar, 1 cm. **l**, IHC assay revealing the expression level of MAFG after silencing MAFG or combined with overexpressing METTL14 in MUM-2B cells. **m**, Representative ORO images of lung and liver tissues

To identify the endogenous critical transcription factor that activates METTL14 and promotes its oncogenic properties, the upstream 2 kb region of the METTL14 transcription start site was examined. Analysis using the UCSC and TCGA databases revealed a positive link to METTL14 level, while the JASPAR online platform predicted specific transcription factors binding to the METTL14 promoter region and their corresponding binding positions and sequences. This led to the identification of MAFG as a potential key factor involved in activating METTL14 transcription (Fig. 5a, b and Figure S5a, b). Notably, examination of the GTEx (normal controls: *n* = 53) and TCGA (UVM: *n* = 80) datasets demonstrated a significant upregulation of MAFG in UVM (Fig. 5c). Clinical validation using immunohistochemical analysis revealed a marked increase in MAFG expression in CM tissues (*n* = 34) compared to normal choroidal tissues (*n* = 10) (Fig. 5d). Western blot analysis confirmed successful MAFG silencing and overexpression in C918 and MUM-2B cells (Figure S5c). Transwell assays showed that silencing MAFG reduced the invasiveness capacity of C918 and MUM-2B cells, while MAFG upregulation markedly elevated the invasion potential in both CM cell types (Figure S5d). Correlation analysis demonstrated a strong positive linear relationship between METTL14 and MAFG level (*R* = 0.675, *P* < 0.001, Fig. 5e). Subsequent qPCR analysis revealed that silencing MAFG substantially reduced METTL14 mRNA levels in MUM-2B cells (Fig. 5f). Correspondingly, MAFG suppression led to a marked decrease in METTL14 protein abundance, while overexpressing MAFG increased METTL14 expression (Fig. 5g). Additionally, luciferase reporter assays revealed that silencing MAFG significantly reduced the transcriptional activity of METTL14 in cells transfected with the wild-type luciferase reporter plasmid compared to the shNC group, while the mutant reporter plasmid exhibited no influence on METTL14 transcription (Fig. 5h).

Rescue experiments revealed that silencing MAFG reduced the invasiveness of cells, an effect that was partially reversed upon METTL14 overexpression (Figure S5e). In vivo rescue experiments showed that the reduction in lung and liver metastasis caused by silencing MAFG could be reversed by METTL14 overexpression (Fig. 5i-k and Figure S5f-h). IHC assays indicated that MAFG expression in lung and liver metastases exhibited substantial reduction in the shMAFG group versus the shNC group, while the shMAFG + METTL14 group exhibited restored MAFG levels similar to the shNC group (Fig. 5l and Figure S5i). ORO staining suggested markedly diminished lipid deposition in lung and liver metastatic foci in the shMAFG group, which was markedly reversed upon METTL14 overexpression (Fig. 5m and Figure S5j). This evidence indicates the elevated expression of METTL14 in CM is induced by MAFG through transcriptional activation.

### SCD1 inhibitor Aramchol exhibits diet-dependent anti-metastatic effects

**Fig. 6:**
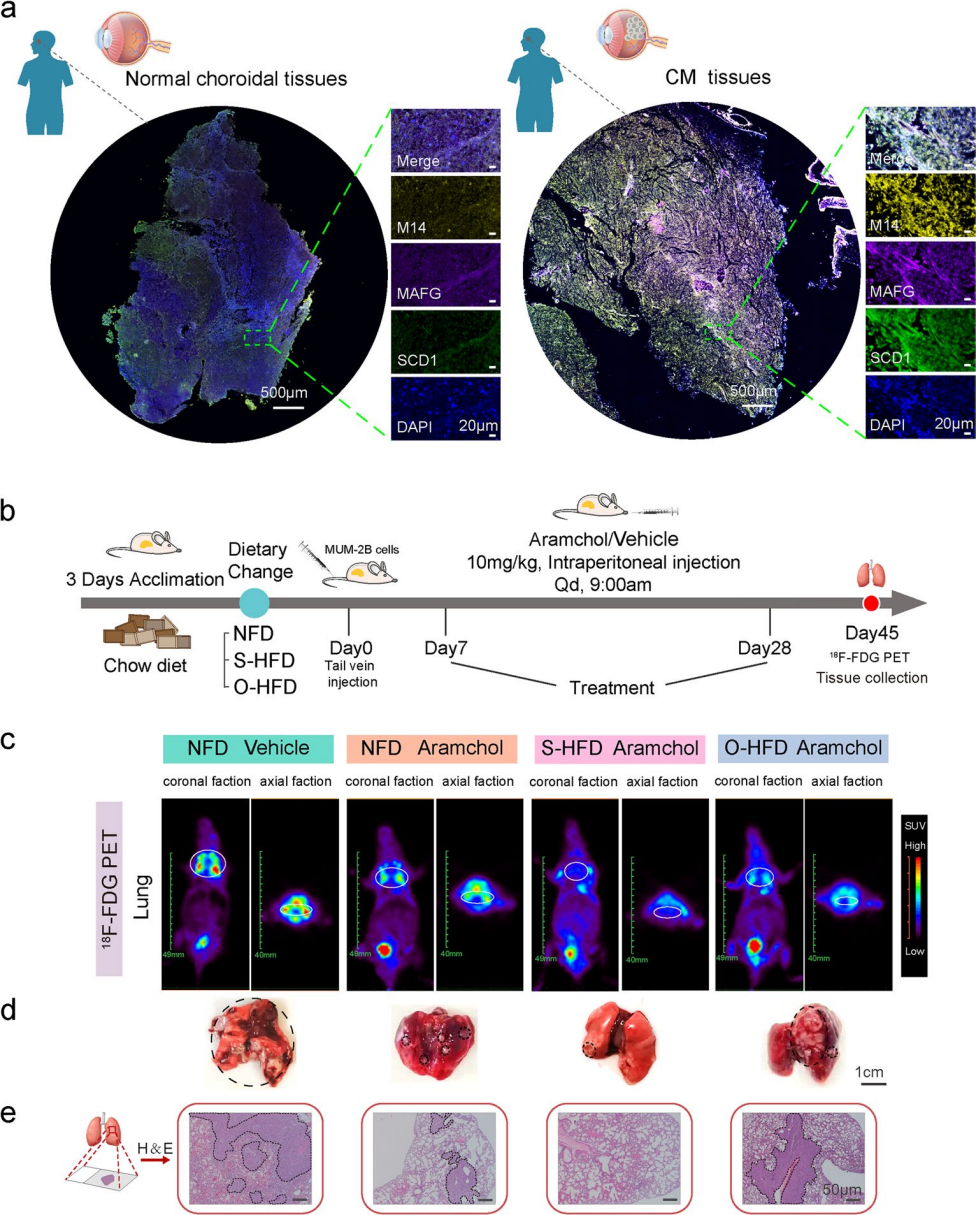
SCD1 inhibitor aramchol exhibits diet-dependent anti-metastatic effects.** a**, Representative multiplexed immunohistochemistry staining images of human normal choroidal tissue and CM tissue samples showing METTL14 (yellow), MAFG (purple), SCD1 (green), and DAPI (blue). **b**, Schematic representation of the therapy schedule. Mice were acclimated to the cages for 3 days, during which they were all fed normal chow (brown-color pellets). Following acclimation, the mice were divided into three dietary groups: normal-fat diet (NFD), high-fat diet rich in stearate (S-HFD), and high-fat diet rich in oleic acid (O-HFD). The interventions began 3 days prior to injection and continued throughout the study (*n* = 5). **c**, Representative coronal and axial sectional PET images showing the uptake of ^18^F-FDG in the lung of mice in four different groups. **d**, Representative images of excised lungs from mice injected with the indicated cells. Scale bar, 1 cm. **e**, Representative H&E staining images of lung tissues

To explore the expression relationship between MAFG, METTL14, and SCD1 in CM, analysis of the TCGA database revealed a significant positive link between MAFG and SCD1 level (*R* = 0.583, Figure S6a). Transwell assays showed that MAFG knockdown reduced the invasive capacity of C918 and MUM-2B cells, an effect rescued by SCD1 overexpression (Figure S6b). Clinical confirmation via mIHC analysis of normal choroidal and CM tissues revealed co-expression of MAFG, METTL14, and SCD1, with markedly higher expression levels in CM tissues (Fig. 6a and Figure S6c). These observations establish the critical role of the MAFG-METTL14-SCD1 axis in promoting CM metastasis. Additionally, the impact of diet switching on experimental metastasis was assessed by analyzing lung metastases. The therapeutic effect of combining the SCD1 inhibitor aramchol with distinct fatty acid-enriched diets on metastatic progression in CM preclinical mouse models was further explored (Fig. 6b). Aramchol, a novel partial inhibitor of SCD1, has shown promise as a treatment for nonalcoholic steatohepatitis (NASH) and was not linked to serious adverse effects in a phase 2b clinical study, unlike other SCD1 inhibitors [43]. Preclinical metastasis models suggested that aramchol alone inhibited tumor metastasis, with the effect further enhanced by feeding mice an S-HFD. In these experiments, the S-HFD group exhibited a more pronounced reduction in metastasis than both the NFD and O-HFD groups. Potent antitumor effects were observed in vivo, attributed to elevated tumor stearate levels from S-HFD feeding and oleate suppression via SCD1 inhibition. Conversely, high oleate intake from the O-HFD markedly weakened this effect (Fig. 6c-e and Figure S6d, e). Under S-HFD conditions, CM metastases displayed an enhanced response to SCD1 inhibitor treatment.

**Fig. 7:**
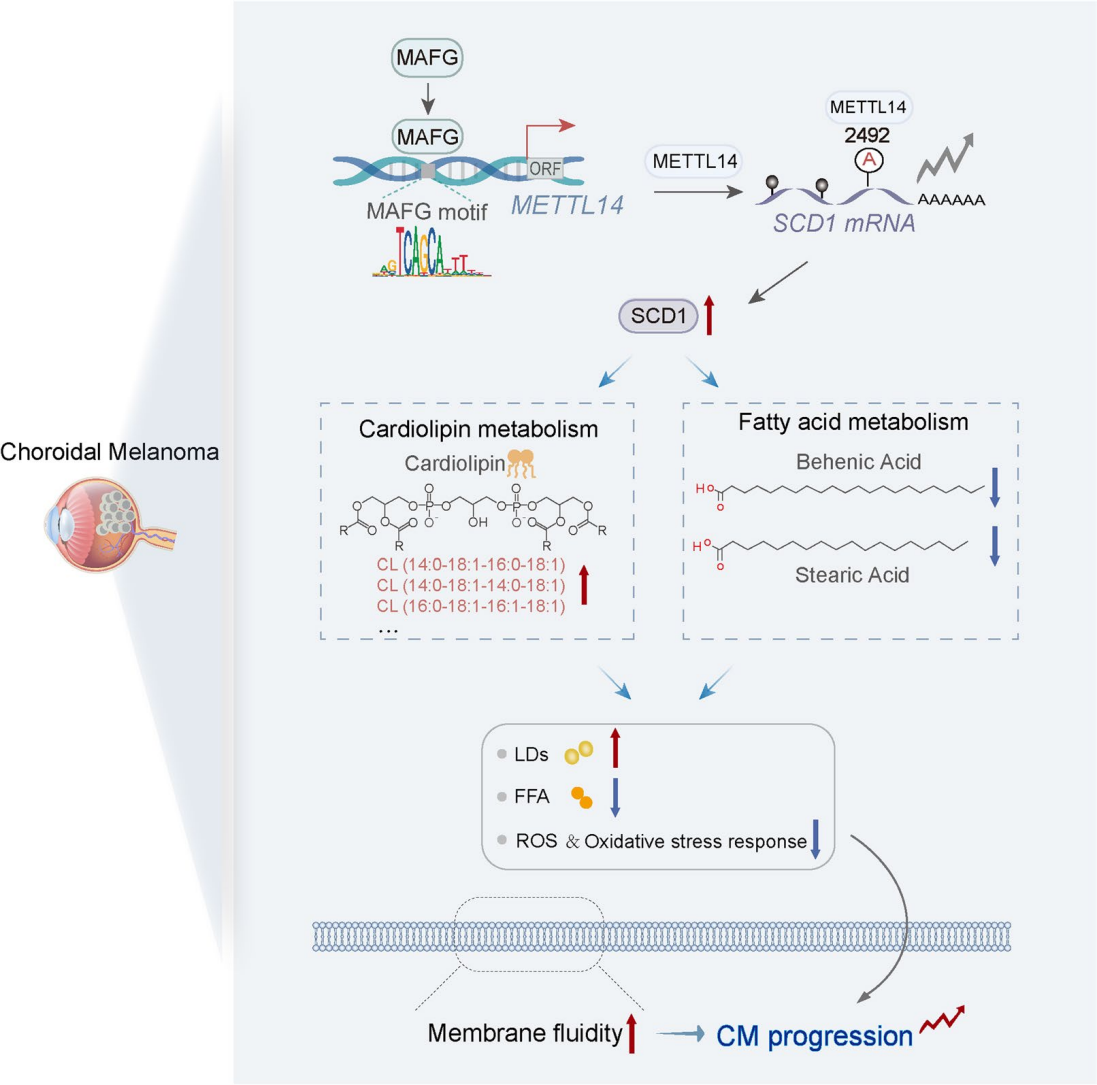
Proposed model showing that the newly identified MAFG-METTL14-SCD1 axis regulates lipid metabolism to mediate distant metastasis of choroidal melanoma

## Discussion

Our study establishes that the MAFG-METTL14-SCD1 axis mediates CM progression through cardiolipin metabolism and fatty acid pathways (Fig. 7). Enhanced cellular membrane fluidity is a fundamental trait that contributes to the metastatic potential of tumors, markedly promoting the invasion and metastasis of cancer cells [6]. This study, for the first time, explores the complex interplay between lipid metabolism, membrane fluidity, invasion, and metastasis. Notably, it reveals that SCD1 promotes cell membrane fluidity *via* the lipid metabolism pathway, thereby promoting CM invasion and metastasis. This work offers a novel theoretical framework and potential strategies for understanding CM metastasis mechanisms.

Our findings provide compelling evidence that abnormal cell membrane fluidity is influenced by key lipids, such as CL, Behenic acid, and Stearic acid. Lipidomic analysis demonstrated that SCD1 suppression resulted in a reduction in the peak areas of CL levels. This decline in CL correlates with a decrease in CM cell membrane fluidity, which serves a pivotal function in tumor invasion and metastasis. It was conclusively shown that a reduction in membrane fluidity markedly impairs the invasive and metastatic capacity of cancer cells. By the same token, if the content of SCD1 increases after exogenous administration of CL, then crosstalk will be formed between CL and SCD1. CL was identified as a critical regulator in tumor initiation, progression, and metastasis [44]. Alterations in cardiolipin metabolism directly affect tumor cell growth and survival [45]. Behenic acid and Stearic acid integrate into the fatty acid chains of glycerophospholipids, enhancing interactions between glycerophospholipid molecules and reducing membrane fluidity [38, 39]. These findings suggest that targeting cell membrane fluidity, cardiolipin, and fatty acid metabolism could provide valuable therapeutic strategies for CM treatment.

In this study, lipidomics analysis demonstrated that downregulation of both SCD1 and METTL14 led to a reduction in the peak areas of CL. This reduction was associated with decreased membrane fluidity and impaired invasion and metastasis capacity. Notably, key lipid metabolism components, Behenic acid and Stearic acid, exhibited elevated expression in both SCD1 and METTL14 silencing groups, suggesting a functional link between SCD1 and METTL14 in modulating CM lipid metabolism. Validation experiments, including Transwell invasion assays, membrane fluidity assessments, electron microscopy of lipid droplets, and nude mouse models for distant organ metastasis, confirmed the pivotal role of METTL14 in CM *via* regulation of SCD1. Mechanistically, this investigation initially revealed that enhanced SCD1 in CM is mediated by m^6^A modification at position 2492 on the *SCD1* mRNA, induced by METTL14 overexpression, thereby facilitating CM lipid metabolism. Lipid metabolism has been found to exhibit a strong link to m^6^A regulation across multiple tumor types. For example, Li et al. demonstrated that METTL14 enhances ANKRD22 mRNA stability and translation in an m^6^A-dependent manner, promoting lipid metabolism in nasopharyngeal carcinoma (NPC) cells through ANKRD22 upregulation [46]. Additionally, m^6^A modulates key genes in metabolic pathways, leading to alterations in lipid metabolism in cancer cells. For example, METTL3-induced m^6^A modification is involved in the upregulation of POU6F2-AS1. Furthermore, upregulated POU6F2-AS1 could tether YBX1 to the FASN promoter to induce transcriptional activation, thus facilitating the growth and lipogenesis of CRC cells [47]. Ultimately, the study identifies, for the first time, that the key lipid metabolism enzyme SCD1 is highly expressed in CM, with this upregulation mediated by m^6^A methylation of its mRNA at the 2492 site by METTL14. This study provides a novel perspective and a solid theoretical foundation for a deeper understanding of CM biological processes.

Transcription factors are critical regulators of human physiology and pathology [48]. Numerous studies have clearly established the central role of the transcription factor MAFG in tumor invasion and metastasis [49–51]. In melanoma cell lines, MAFG is markedly upregulated, highlighting its essential involvement in melanoma progression [52]. We demonstrated for the first time that MAFG is highly expressed in choroidal melanoma. Deng et al. reported the pronounced expression of MAFG in fibrotic liver tissues and activated hepatic stellate cells, where MAFG directly interacts with the promoter region of LCN2, activating its transcription and influencing iron ion metabolism in hepatic stellate cells. This interaction serves a pivotal function in preventing iron-induced cell death and promoting liver fibrosis progression [53]. Our novel findings further highlight the direct binding of MAFG to METTL14, revealing that MAFG specifically binds to the promoter region of METTL14, and its heightened expression in choroidal melanoma is dependent on MAFG’s recognition of a critical motif, which triggers its specific activation.

This study demonstrates that the high invasiveness and metastasis of CM are linked to enhanced membrane fluidity mediated by SCD1. Inhibition of SCD1 results in increased lipid saturation, depleted lipid droplets, reduced membrane fluidity, elevated ROS levels, and induced oxidative stress. Specifically, the elevated ROS generated by SCD1 inhibition damages mitochondria and activates the pro-apoptotic Bcl-2-associated X protein (BAX); activated BAX then promotes cytochrome c release, which in turn initiates the mitochondrial apoptotic pathway and activates caspase-9, ultimately leading to tumor cell death and thereby suppressing tumor metastasis [54, 55]. Focusing on clinical translation, the targeting of SCD1 activity is prioritized. In contrast to other SCD1 inhibitors, aramchol does not cause severe adverse effects. The efficacy and safety profile of aramchol has been assessed in a randomized, double-blind, placebo-controlled phase 2b study involving NASH patients [43]. Preclinical evidence indicates that combining aramchol with S-HFD markedly reduces CM metastasis to distant organs in nude mouse models, highlighting its potential for clinical application. The enhanced anti-metastatic efficacy of the S-HFD/aramchol combination arises from dual metabolic disruptions: the accumulation of dietary-derived stearate and the oleate deficiency induced by SCD1 inhibition in nude mouse models. Our findings also demonstrate that the MAFG-METTL14-SCD1 axis drives CM progression through cardiolipin metabolism and fatty acid pathways. Notably, this study definitively establishes the pro-cancer role of METTL14 in CM, despite the ongoing controversy surrounding its function in ocular melanoma. Some studies suggest that METTL14 may act as a tumor suppressor in ocular melanoma, highlighting the complexities and unresolved questions surrounding m^6^A regulation. These inconsistencies emphasize the need for further investigation into the role of m^6^A and its associated factors, driving the development of research on RNA methylation to deeper levels.

In conclusion, comprehensive in vivo and in vitro experiments, coupled with clinical sample analysis, confirm that the MAFG-METTL14-SCD1 axis promotes choroidal melanoma cell metastasis by modulating cardiolipin and fatty acid pathways. Through in-depth clinical validation based on precious choroidal tissues, we have been able to generate more reliable and detailed results. This study reveals a novel mechanism linking lipid metabolism to membrane fluidity and CM metastasis, highlighting the therapeutic potential of inhibiting SCD1 function in cancer treatment. Moreover, the combination of the SCD1 inhibitor aramchol and S-HFD identified in this study may offer a promising strategy. These findings provide a solid theoretical foundation for upcoming clinical trials.

## Supplementary Information

Supplementary Material 1.

## Data Availability

No datasets were generated or analysed during the current study.

## Abbreviations

BCA: bicinchoninic acid
CC: colorectal cancer
CFA: colony formation assay
CIDEC: cell death-inducing DFF45-like effector c
CM: choroidal melanoma
EC: endometrial cancer
ERRα: estrogen-related receptor α
FISH: fluorescence in situ hybridization
GPs: glycerophospholipids
H&E: hematoxylin and eosin
HCA: hierarchical clustering analysis
HCC: hepatocellular carcinoma
HFD: high-fat diet
HRP: horseradish peroxidase
IgG: immunoglobulin G
IHC: immunohistochemistry
LPCAT1: lysophosphatidylcholine acyltransferase 1
m^6^A: N6-methyladenosine
MeRIP: methylated RNA immunoprecipitation
mIHC: multiplexed immunohistochemistry
MUFA: monounsaturated fatty acids
NASH: nonalcoholic steatohepatitis
NFD: normal-fat diet
NPC: nasopharyngeal carcinoma
OD: optical density
O-HFD: high-fat diet enriched with oleic acid
ORO: Oil Red O
PCA: principal component analysis
PLS-DA: partial least squares discriminant analysis
PMSF: phenylmethylsulfonyl fluoride
PVF: polyvinyl formal
qPCR: quantitative polymerase chain reaction
ROS: reactive oxygen species
RT-PCR: reverse transcription polymerase chain reaction
SCD1: stearoyl-CoA desaturase 1
SFA-PC: saturated fatty acyl phosphatidylcholine
SFAs: saturated fatty acids
S-HFD: stearate-supplemented high-fat diet
SREBPs: sterol regulatory element-binding proteins
TEM: transmission electron microscopy
TFEB: transcription factor EB
UM: uveal melanoma
WT: wild-type

## References

[1] Coupland SE, Lake SL, Zeschnigk M, Damato BE. Molecular pathology of uveal melanoma. Eye (Lond). 2013;27(2):230–42. 23222563 10.1038/eye.2012.255 PMC3574255

[2] Carvajal RD, Sacco JJ, Jager MJ, Eschelman DJ, Olofsson Bagge R, Harbour JW, et al. Advances in the clinical management of uveal melanoma. Nat Rev Clin Oncol. 2023;20(2):99–115. 36600005 10.1038/s41571-022-00714-1

[3] Rantala ES, Hernberg MM, Piperno-Neumann S, Grossniklaus HE, Kivelä TT. Metastatic uveal melanoma: the final frontier. Prog Retin Eye Res. 2022;90:101041. 34999237 10.1016/j.preteyeres.2022.101041

[4] Kujala E, Mäkitie T, Kivelä T. Very long-term prognosis of patients with malignant uveal melanoma. Invest Ophthalmol Vis Sci. 2003;44(11):4651–9. 14578381 10.1167/iovs.03-0538

[5] Jensen OA. Malignant melanomas of the human uvea: 25-year follow-up of cases in Denmark, 1943–1952. Acta Ophthalmol (Copenh). 1982;60(2):161–82. 7136531 10.1111/j.1755-3768.1982.tb08371.x

[6] Zhao W, Prijic S, Urban BC, Tisza MJ, Zuo Y, Li L, et al. Candidate antimetastasis drugs suppress the metastatic capacity of breast cancer cells by reducing membrane fluidity. Cancer Res. 2016;76(7):2037–49. 26825169 10.1158/0008-5472.CAN-15-1970 PMC8491548

[7] Lorent JH, Levental KR, Ganesan L, Rivera-Longsworth G, Sezgin E, Doktorova M, et al. Plasma membranes are asymmetric in lipid unsaturation, packing and protein shape. Nat Chem Biol. 2020;16(6):644–52. 32367017 10.1038/s41589-020-0529-6 PMC7246138

[8] Sakuragi T, Nagata S. Regulation of phospholipid distribution in the lipid bilayer by flippases and scramblases. Nat Rev Mol Cell Biol. 2023;24(8):576–96. 37106071 10.1038/s41580-023-00604-z PMC10134735

[9] Martin-Perez M, Urdiroz-Urricelqui U, Bigas C, Benitah SA. The role of lipids in cancer progression and metastasis. Cell Metab. 2022;34(11):1675–99. 36261043 10.1016/j.cmet.2022.09.023

[10] Vogel FCE, Chaves-Filho AB, Schulze A. Lipids as mediators of cancer progression and metastasis. Nat Cancer. 2024;5(1):16–29. 38273023 10.1038/s43018-023-00702-z

[11] Zhang L, Zhao J, Lam SM, Chen L, Gao Y, Wang W, et al. Low-input lipidomics reveals lipid metabolism remodelling during early mammalian embryo development. Nat Cell Biol. 2024;26(2):278–93. 38302721 10.1038/s41556-023-01341-3

[12] Wang Y, Hu M, Cao J, Wang F, Han JR, Wu TW et al. ACSL4 and polyunsaturated lipids support metastatic extravasation and colonization. Cell. 2025;188(2):412–429. 10.1016/j.cell.2024.10.047 39591965

[13] Mao X, Lei H, Yi T, Su P, Tang S, Tong Y, et al. Lipid reprogramming induced by the TFEB-ERRα axis enhanced membrane fluidity to promote EC progression. J Exp Clin Cancer Res. 2022;41(1):28. 35045880 10.1186/s13046-021-02211-2 PMC8767755

[14] Liu Q, Zhang X, Qi J, Tian X, Dovjak E, Zhang J et al. Comprehensive profiling of lipid metabolic reprogramming expands precision medicine for HCC. Hepatology. 2025;81(4):1164–1180. 10.1097/HEP.0000000000000962 PMC11902616 38899975

[15] Belleri M, Paganini G, Coltrini D, Ronca R, Zizioli D, Corsini M, et al. β-Galactosylceramidase promotes melanoma growth via modulation of ceramide metabolism. Cancer Res. 2020;80(22):5011–23. 32998995 10.1158/0008-5472.CAN-19-3382

[16] Janneh AH, Kassir MF, Atilgan FC, Lee HG, Sheridan M, Oleinik N, et al. Crosstalk between pro-survival sphingolipid metabolism and complement signaling induces inflammasome-mediated tumor metastasis. Cell Rep. 2022;41(10):111742. 36476873 10.1016/j.celrep.2022.111742 PMC9791981

[17] Sen U, Coleman C, Sen T. Stearoyl coenzyme A desaturase-1: multitasker in cancer, metabolism, and ferroptosis. Trends Cancer. 2023;9(6):480–9. 37029018 10.1016/j.trecan.2023.03.003

[18] Luis G, Godfroid A, Nishiumi S, Cimino J, Blacher S, Maquoi E, et al. Tumor resistance to ferroptosis driven by Stearoyl-CoA Desaturase-1 (SCD1) in cancer cells and fatty acid biding Protein-4 (FABP4) in tumor microenvironment promote tumor recurrence. Redox Biol. 2021;43:102006. 34030117 10.1016/j.redox.2021.102006 PMC8163990

[19] Koeberle A, Löser K, Thürmer M. Stearoyl-CoA desaturase-1 and adaptive stress signaling. Biochim Biophys Acta. 2016;1861(11):1719–26. 27550503 10.1016/j.bbalip.2016.08.009

[20] Liu HH, Xu Y, Li CJ, Hsu SJ, Lin XH, Zhang R, et al. An SCD1-dependent mechanoresponsive pathway promotes HCC invasion and metastasis through lipid metabolic reprogramming. Mol Ther. 2022;30(7):2554–67. 35358687 10.1016/j.ymthe.2022.03.015 PMC9263248

[21] Che G, Wang W, Wang J, He C, Yin J, Chen Z, et al. Sulfotransferase SULT2B1 facilitates colon cancer metastasis by promoting SCD1-mediated lipid metabolism. Clin Transl Med. 2024;14(2):e1587. 38372484 10.1002/ctm2.1587 PMC10875708

[22] Yang Y, Cai J, Yang X, Wang K, Sun K, Yang Z, et al. Dysregulated m6A modification promotes lipogenesis and development of non-alcoholic fatty liver disease and hepatocellular carcinoma. Mol Ther. 2022;30(6):2342–53. 35192934 10.1016/j.ymthe.2022.02.021 PMC9171149

[23] Xia W, Veeragandham P, Cao Y, Xu Y, Rhyne TE, Qian J, et al. Obesity causes mitochondrial fragmentation and dysfunction in white adipocytes due to RalA activation. Nat Metab. 2024;6(2):273–89. 38286821 10.1038/s42255-024-00978-0 PMC10896723

[24] Göransson O, Kopietz F, Rider MH. Metabolic control by AMPK in white adipose tissue. Trends Endocrinol Metab. 2023;34(11):704–17. 37673765 10.1016/j.tem.2023.08.011

[25] He Y, B’Nai Taub A, Yu L, Yao Y, Zhang R, Zahr T, et al. PPARγ acetylation orchestrates adipose plasticity and metabolic rhythms. Adv Sci (Weinh). 2023;10(2):e2204190. 36394167 10.1002/advs.202204190 PMC9839851

[26] Wang T, Kong S, Tao M, Ju S. The potential role of RNA N6-methyladenosine in cancer progression. Mol Cancer. 2020;19(1):88. 32398132 10.1186/s12943-020-01204-7 PMC7216508

[27] Sun B, Wang G, Chen G, Zhang Y, Yang R, Hua H et al. GNAO1 overexpression promotes neural differentiation of glioma stem-like cells and reduces tumorigenicity through TRIM21/CREB/HES1 axis. Oncogene. 2025;44(7):450–461. 10.1038/s41388-024-03234-7 39580518

[28] Gao ZX, Li CL, Zhang H, Zhang GH, Zhang Y, Guo XY et al. LINC00882, transcriptionally activated by CEBP-β and post-transcriptionally stabilized by METTL14-mediated m(6)A modification, exerts tumorigenesis by promoting PABPC1-mediated stabilization of ELK3 mRNA. Oncogene. 2025;44(6):363–377. 10.1038/s41388-024-03225-8 39551868

[29] Jia R, Chai P, Wang S, Sun B, Xu Y, Yang Y, et al. m(6)A modification suppresses ocular melanoma through modulating HINT2 mRNA translation. Mol Cancer. 2019;18(1):161. 31722709 10.1186/s12943-019-1088-x PMC6854757

[30] Zhang X, Zhang X, Liu T, Zhang Z, Piao C, Ning H. METTL14 promotes migration and invasion of choroidal melanoma by targeting RUNX2 mRNA via m6A modification. J Cell Mol Med. 2022;26(22):5602–13. 36264762 10.1111/jcmm.17577 PMC9667526

[31] Zhang B, Jiang H, Dong Z, Sun A, Ge J. The critical roles of m6A modification in metabolic abnormality and cardiovascular diseases. Genes Dis. 2021;8(6):746–58. 34522705 10.1016/j.gendis.2020.07.011 PMC8427257

[32] Zhao L, Guo J, Xu S, Duan M, Liu B, Zhao H et al. Abnormal changes in metabolites caused by m(6)A methylation modification: the leading factors that induce the formation of immunosuppressive tumor microenvironment and their promising potential for clinical application. J Adv Res. 2025;70:159–186. 10.1016/j.jare.2024.04.016 PMC11976433 38677545

[33] Zhou Q, Liu X, Lu H, Li N, Meng J, Huang J, et al. m6A-methylase METTL3 promotes retinal angiogenesis through modulation of metabolic reprogramming in RPE cells. J Neuroinflammation. 2024;21(1):289. 39506758 10.1186/s12974-024-03279-1 PMC11539582

[34] Wang C, Tanizawa H, Hill C, Havas A, Zhang Q, Liao L, et al. METTL3-mediated chromatin contacts promote stress granule phase separation through metabolic reprogramming during senescence. Nat Commun. 2024;15(1):5410. 38926365 10.1038/s41467-024-49745-5 PMC11208586

[35] Peng H, Chen B, Wei W, Guo S, Han H, Yang C, et al. N(6)-methyladenosine (m(6)A) in 18S rRNA promotes fatty acid metabolism and oncogenic transformation. Nat Metab. 2022;4(8):1041–54. 35999469 10.1038/s42255-022-00622-9

[36] Chen A, Chen X, Cheng S, Shu L, Yan M, Yao L, et al. FTO promotes SREBP1c maturation and enhances CIDEC transcription during lipid accumulation in HepG2 cells. Biochim Biophys Acta Mol Cell Biol Lipids. 2018;1863(5):538–48. 29486327 10.1016/j.bbalip.2018.02.003

[37] Chen H, Liu C, Cui S, Xia Y, Zhang K, Cheng H et al. Intermittent fasting triggers interorgan communication to suppress hair follicle regeneration. Cell. 2025;188(1):157–174. 10.1016/j.cell.2024.11.004 39674178

[38] Gangopadhyay S, Vijayan VK, Bansal SK. Lipids of erythrocyte membranes of COPD patients: a quantitative and qualitative study. Copd. 2012;9(4):322–31. 22497562 10.3109/15412555.2012.668581

[39] Gew LT, Misran M. Interaction between C18 fatty acids and DOPE PEG2000 in Langmuir monolayers: effect of degree of unsaturation. J Biol Phys. 2017;43(3):397–414. 28752254 10.1007/s10867-017-9459-2 PMC6104901

[40] Wang Y, Zhou X, Lei Y, Chu Y, Yu X, Tong Q, et al. NNMT contributes to high metastasis of triple negative breast cancer by enhancing PP2A/MEK/ERK/c-Jun/ABCA1 pathway mediated membrane fluidity. Cancer Lett. 2022;547:215884. 35988817 10.1016/j.canlet.2022.215884

[41] Kurtipek E, Çayci M, Düzgün N, Esme H, Terzi Y, Bakdik S, et al. (18)F-FDG PET/CT mean SUV and metabolic tumor volume for mean survival time in non-small cell lung cancer. Clin Nucl Med. 2015;40(6):459–63. 25742234 10.1097/RLU.0000000000000740

[42] Nair VS, Barnett PG, Ananth L, Gould MK. PET scan 18F-fluorodeoxyglucose uptake and prognosis in patients with resected clinical stage IA non-small cell lung cancer. Chest. 2010;137(5):1150–6. 20038738 10.1378/chest.09-2356

[43] Ratziu V, de Guevara L, Safadi R, Poordad F, Fuster F, Flores-Figueroa J, et al. Aramchol in patients with nonalcoholic steatohepatitis: a randomized, double-blind, placebo-controlled phase 2b trial. Nat Med. 2021;27(10):1825–35. 34621052 10.1038/s41591-021-01495-3 PMC12165723

[44] Bian H, Ma D, Pan F, Zhang X, Xin K, Zhang X, et al. Cardiolipin-Targeted NIR-II fluorophore causes avalanche effects for Re-Engaging cancer apoptosis and inhibiting metastasis. J Am Chem Soc. 2022;144(49):22562–73. 36445324 10.1021/jacs.2c08602

[45] Guri Y, Colombi M, Dazert E, Hindupur SK, Roszik J, Moes S, et al. mTORC2 promotes tumorigenesis via lipid synthesis. Cancer Cell. 2017;32(6):807–e2312. 29232555 10.1016/j.ccell.2017.11.011

[46] Li L, Tang Q, Ge J, Wang D, Mo Y, Zhang Y, et al. METTL14 promotes lipid metabolism reprogramming and sustains nasopharyngeal carcinoma progression via enhancing m(6)A modification of ANKRD22 mRNA. Clin Transl Med. 2024;14(7):e1766. 39021049 10.1002/ctm2.1766 PMC11255023

[47] Jiang T, Qi J, Xue Z, Liu B, Liu J, Hu Q, et al. The m(6)A modification mediated-lncRNA POU6F2-AS1 reprograms fatty acid metabolism and facilitates the growth of colorectal cancer via upregulation of FASN. Mol Cancer. 2024;23(1):55. 38491348 10.1186/s12943-024-01962-8 PMC10943897

[48] Fuxman Bass JI, Sahni N, Shrestha S, Garcia-Gonzalez A, Mori A, Bhat N, et al. Human gene-centered transcription factor networks for enhancers and disease variants. Cell. 2015;161(3):661–73. 25910213 10.1016/j.cell.2015.03.003 PMC4409666

[49] Deng Y, Lu L, Zhang H, Fu Y, Liu T, Chen Y. The role and regulation of Maf proteins in cancer. Biomark Res. 2023;11(1):17. 36750911 10.1186/s40364-023-00457-w PMC9903618

[50] Vera-Puente O, Rodriguez-Antolin C, Salgado-Figueroa A, Michalska P, Pernia O, Reid BM, et al. MAFG is a potential therapeutic target to restore chemosensitivity in cisplatin-resistant cancer cells by increasing reactive oxygen species. Transl Res. 2018;200:1–17. 30053382 10.1016/j.trsl.2018.06.005 PMC7787305

[51] Liu T, Yang H, Fan W, Tu J, Li TWH, Wang J, et al. Mechanisms of MAFG dysregulation in cholestatic liver injury and development of liver cancer. Gastroenterology. 2018;155(2):557–e7114. 29733835 10.1053/j.gastro.2018.04.032 PMC6067975

[52] Vera O, Martinez M, Soto-Vargas Z, Wang K, Xu X, Ruiz-Buceta S et al. The small MAF transcription factor MAFG co-opts MITF to promote melanoma progression. BioRxiv. [Preprint]. 2024 Sep

[53] Deng Y, Lu L, Zhu D, Zhang H, Fu Y, Tan Y, et al. MafG/MYH9-LCN2 axis promotes liver fibrosis through inhibiting ferroptosis of hepatic stellate cells. Cell Death Differ. 2024;31(9):1127–39. 38871948 10.1038/s41418-024-01322-5 PMC11369194

[54] Green DR. Nonapoptotic cell death pathways. Cold Spring Harb Perspect Biol. 2022;14(11):a041079. 36319068 10.1101/cshperspect.a041079 PMC9620857

[55] Schafer ZT, Kornbluth S. The apoptosome: physiological, developmental, and pathological modes of regulation. Dev Cell. 2006;10(5):549–61. 16678772 10.1016/j.devcel.2006.04.008

