# Supplementary material for: Novel MAFG-METTL14-SCD1 axis regulates lipid metabolism mediating choroidal melanoma distant metastasis
Source: J Exp Clin Cancer Res. 2025 Nov 29;44:334. doi: 10.1186/s13046-025-03595-1 (PMC12751620; doi:10.1186/s13046-025-03595-1)
Supplement: Supplementary file 1 — Supplementary Material 1. [file 13046_2025_3595_MOESM1_ESM.pdf]

Additional file 1

This file includes:

Fig S1. METTL14 promotes proliferation and metastasis of CM cells.

Fig S2. Increased levels of SCD1 associate with poor prognosis in UVM.

Fig S3. SCD1 promotes invasion of CM cells *in vitro*.

Fig S4. The METTL14-SCD1 signal axis promotes CM invasion and metastasis.

Fig S5. The MAFG-METTL14 signaling axis promotes CM invasion and metastasis.

Fig S6. Role of MAFG and SCD1 in CM invasion and metastasis.

Table S1. Patient demographics

Table S2. Short hairpin RNAs (shRNAs) sequence against METTL14

Table S3. Short hairpin RNAs (shRNAs) sequence against SCD1

Table S4. Short hairpin RNAs (shRNAs) sequence against MAFG

Table S5. Primers used in qRT-PCR assays

Table S6. Probes used in FISH assays

Table S7. Primers used in MeRIP assays

Additional file 1

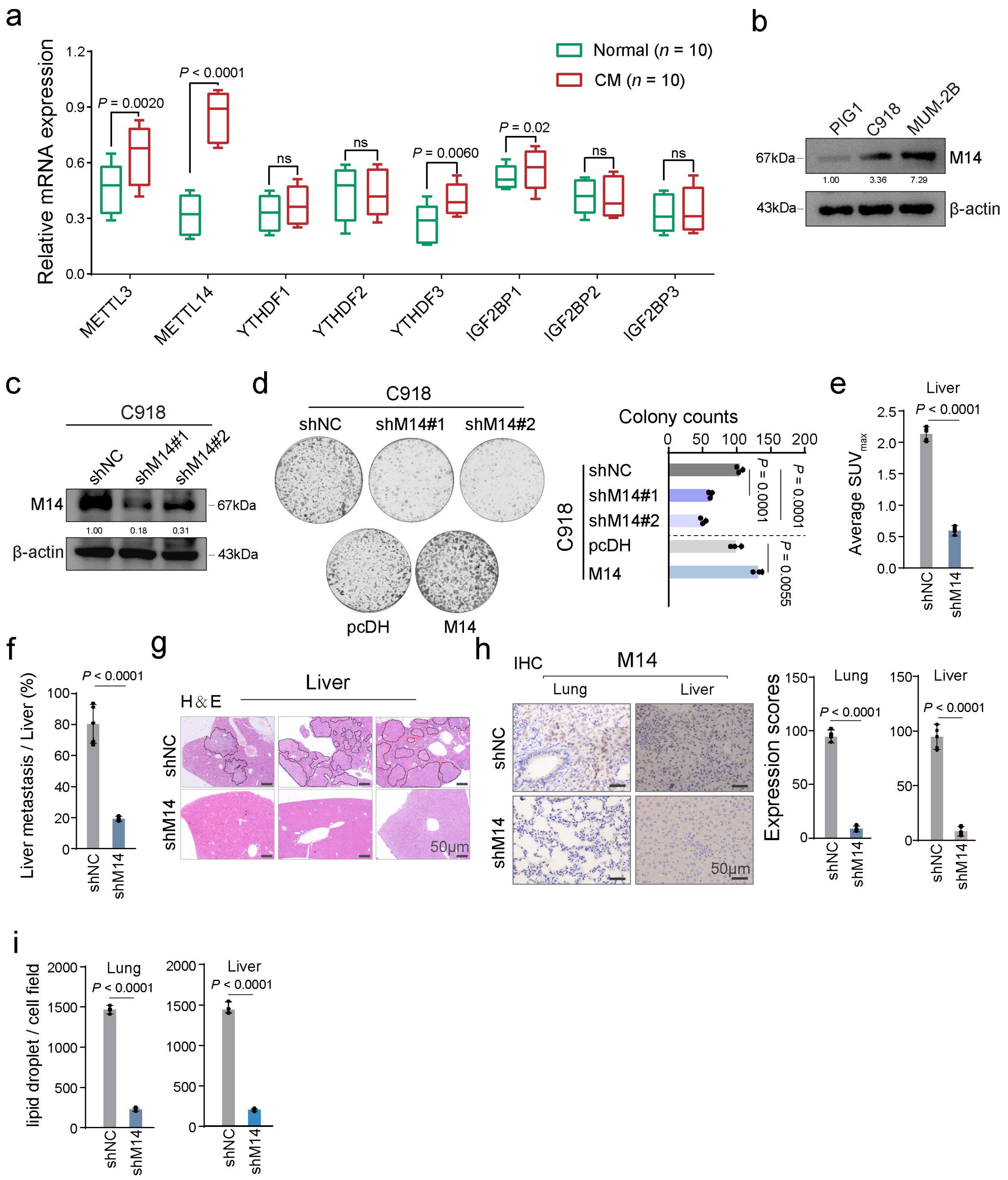

Fig S1. METTL14 promotes proliferation and metastasis of CM cells.

a, Relative mRNA levels of METTL3, METTL14, YTHDF1/2/3 and IGF2BP1/2/3 in CM tissues (n = 10) and normal choroidal tissues (n = 10) detected by qRT-PCR assay. **b,** Representative western blot images of METTL14 in a normal melanocyte cell line (PIG1) and choroidal melanoma cells. **c,** Representative western blot images showing METTL14 expression in CM cells after interfering with METTL14 expression. **d,** Cell proliferation assessed by colony formation assay in METTL14 knockdown or overexpression CM cell lines. **e,** Glucose uptake in the tumor was evaluated by the average SUVmax. **f,** Analysis of liver metastases tumors in the respective groups. **g,** Representative images of Haematoxylin and eosin (H&E) staining of liver tissues. **h,** Immunohistochemical analysis of lung and liver tissues from mice with anti-METTL14 antibody in two different groups (shNC, shMETTL14, n = 5 for each). Scale bar, 50 μm. **i,** The histogram shows the quantification of lipid droplet area per cell field.

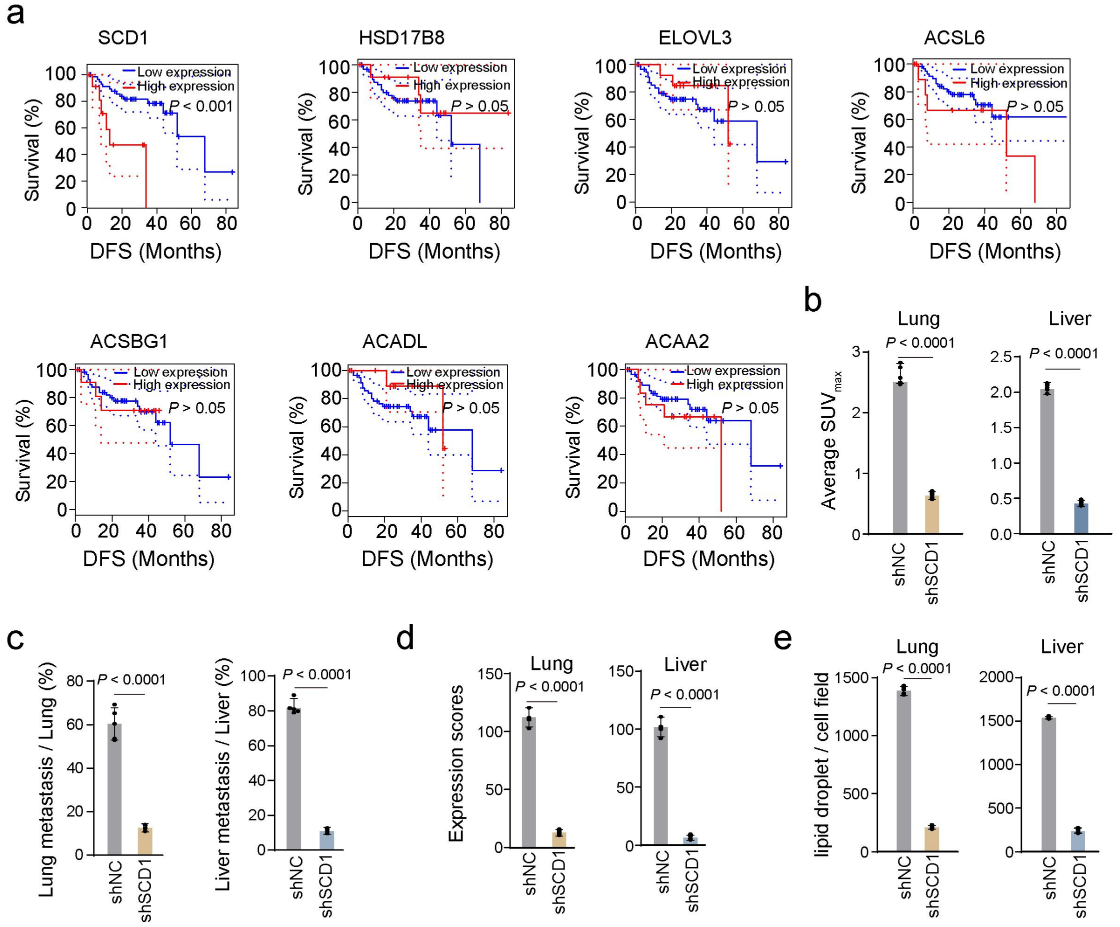

Fig S2. Increased levels of SCD1 associate with poor prognosis in UVM.

a, Kaplan-Meier analysis showing the correlation between the expression of SCD1, HSD17B8, ELOVL3, ACSL6, ACSBG1, ACADL, ACAA2, and Disease-Free Survival in TCGA-uveal melanoma patients. **b,** Glucose uptake in the tumor was evaluated by the average $SUV_{max.}$ **c,** Analysis of lung and liver metastases tumors in the respective groups. **d,** The histogram presents the expression scores from IHC analysis. **e,** The histogram shows the quantification of lipid droplet area per cell field.

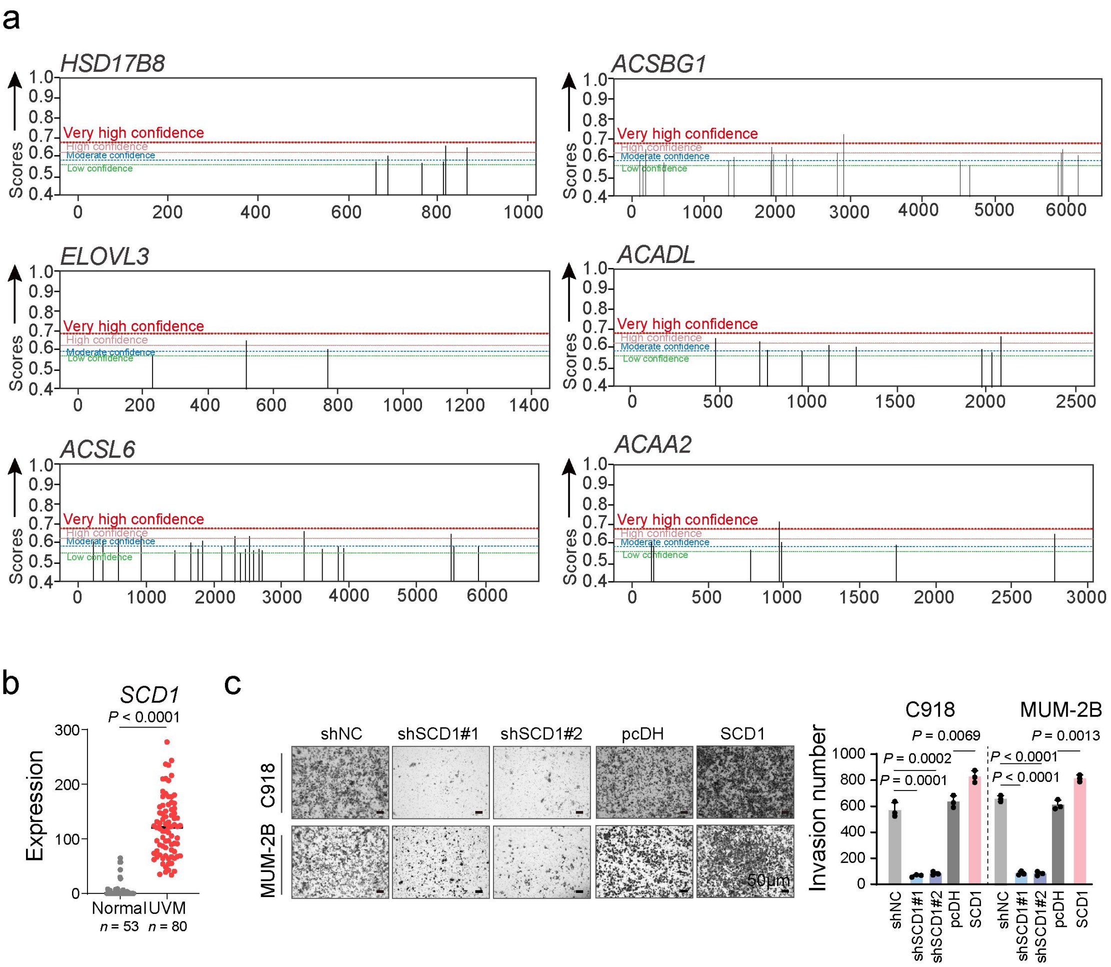

Fig S3. SCD1 promotes invasion of CM cells *in vitro*.

a, The SRAMP (http://www.cuilab.cn/sramp) predicts the distribution of high-confidence m^{6}A methylation sites and scores for HSD17B8, ELOVL3, ACSL6, ACSBG1, ACADL, and ACAA2 mRNA. **b,** The GTEx and TCGA databases showing the differential expression of SCD1 in normal individuals and patients with UVM. GTEx (Normal: n = 53), TCGA (UVM: n = 80). **c,** Cell invasiveness detected by invasion assay.

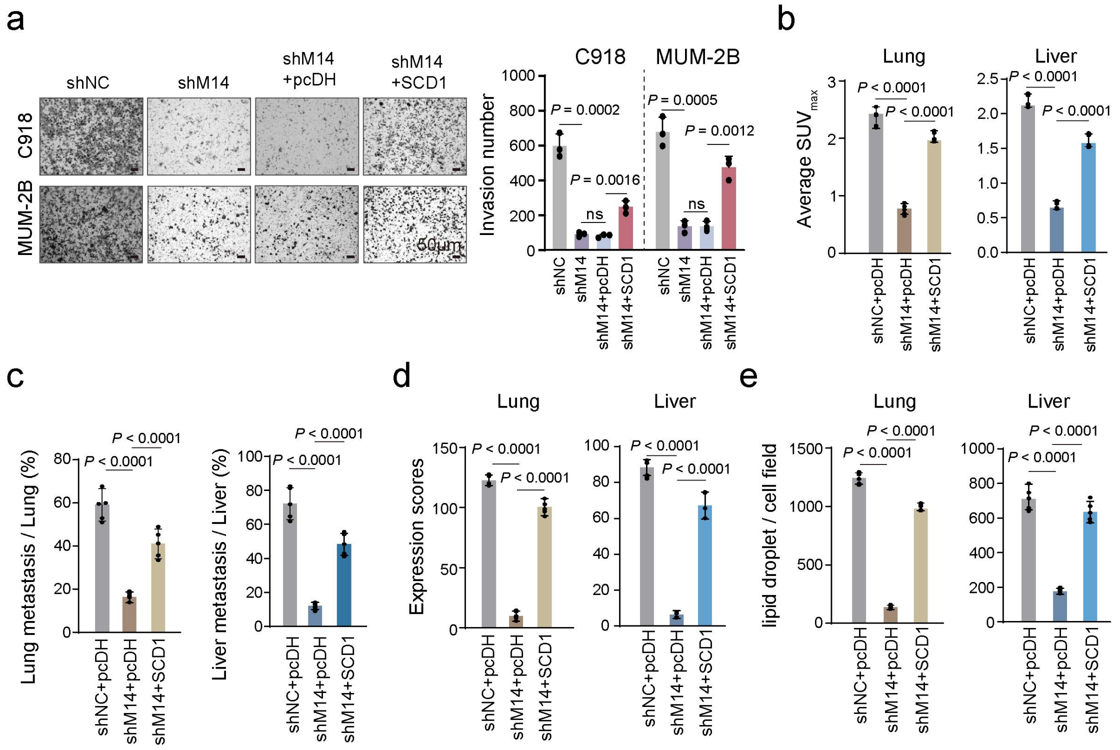

Fig S4. The METTL14-SCD1 signal axis promotes CM invasion and metastasis.

a, Invasion assay used to detect the invasiveness of cells with the indicated treatments. **b,** Glucose uptake in the tumor was evaluated by the average SUV_{max}. **c,** Analysis of lung and liver metastases tumors in the respective groups. **d,** The histogram presents the expression scores from IHC analysis. **e,** The histogram shows the quantification of lipid droplet area per cell field.

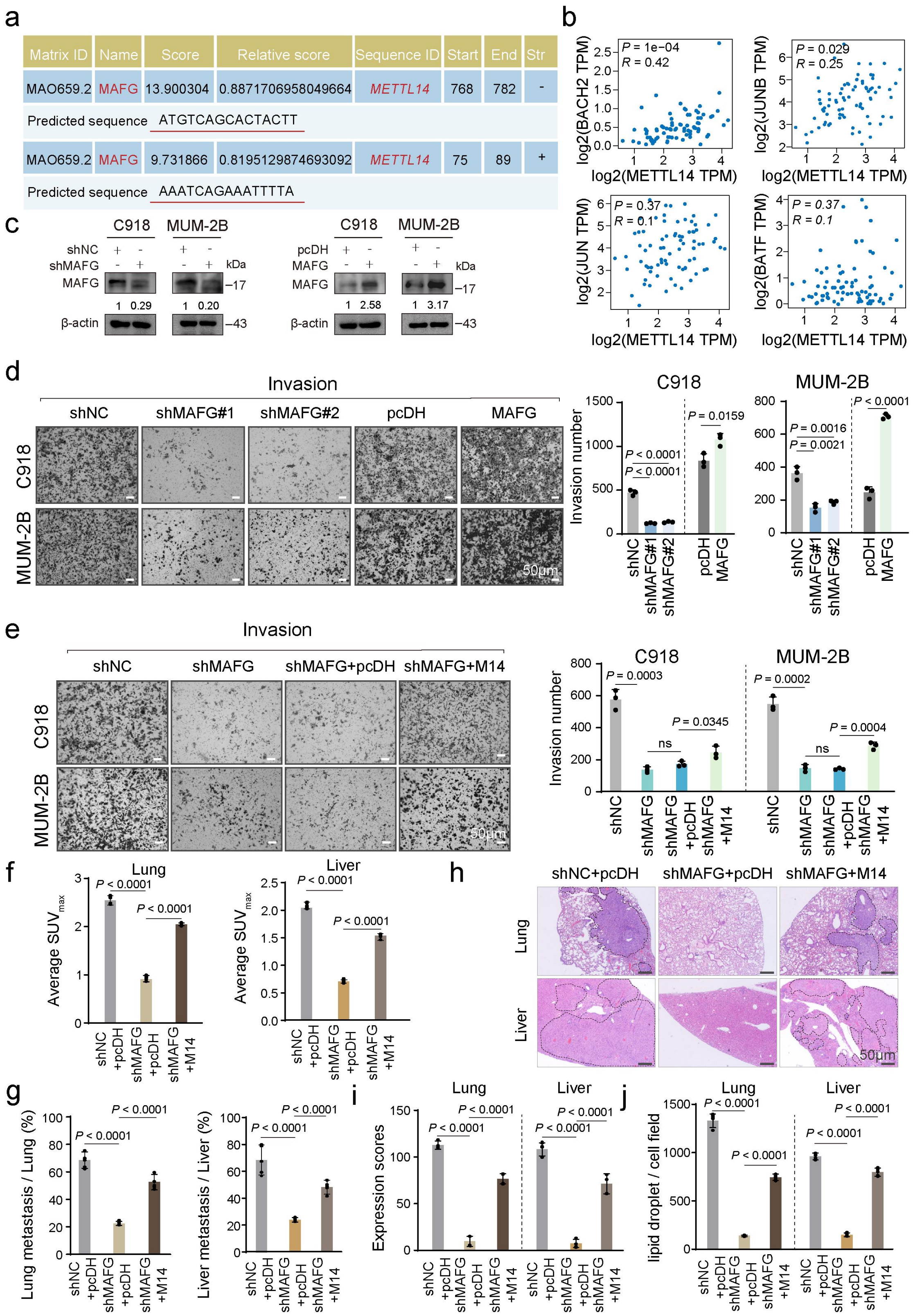

Fig S5. The MAFG-METTL14 signaling axis promotes CM invasion and metastasis.

a, The sites and scores of MAFG in regulating METTL14. **b,** The linear correlation relationship between METTL14 expression and the expression of BACH2, JUNB, JUN, and BATF. **c,** Representative western blot images showing MAFG expression in CM cells after interfering with MAFG expression. **d,** Transwell assays determining the effects of MAFG on invasion capability in

C918 and MUM-2B cells. **e,** Transwell assay demonstrating the effects of MAFG and METTL14 on CM cell invasion. Overexpression of METTL14 partially reversed the reduction in cell invasion caused by decreased MAFG expression. **f,** Glucose uptake in the tumor was evaluated by the average $SUV_{max.}$ **g,** Analysis of lung and liver metastases tumors in the respective groups. **h,** H&E staining results are shown. **i,** The histogram presents the expression scores from IHC analysis. **j,** The histogram shows the quantification of lipid droplet area per cell field.

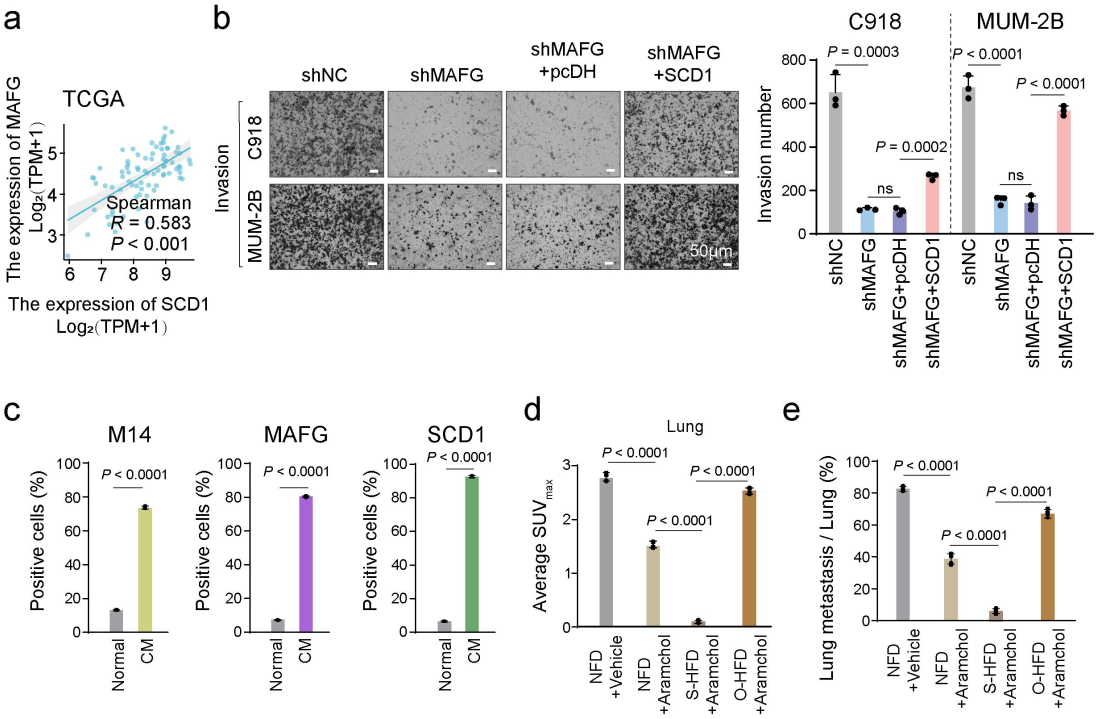

Fig S6. Role of MAFG and SCD1 in CM invasion and metastasis.

a, The linear correlation relationship between SCD1 expression and MAFG expression. **b,** Transwell assay demonstrates the effects of MAFG and SCD1 on the invasion capability of CM cells. **c,** Statistical analysis of the number of METTL14, MAFG, and SCD1 positive cells in normal choroidal tissue and CM tissue samples (n = 5). **d,** Glucose uptake in the tumor was evaluated by the average $SUV_{max.}$ **e,** Analysis of lung metastases tumors in the respective groups.

Table S1. Patient demographics

	**Normal tissue (*n* = 28)**	**Tumor tissue (*n* = 36)**
Gender	Male 10; Female 18	Male 17; Female 19
Age (y)	40 ±20	64 ± 9

Table S2. Short hairpin RNAs (shRNAs) sequence against METTL14

shRNA	Sequence (5'-3')
METTL14-Homo (shRNA#1)	GCATTGGTGCCGTGTTAAATA
METTL14-Homo (shRNA#2)	GCTAAAGGATGAGTTAATAGC

Table S3. Short hairpin RNAs (shRNAs) sequence against SCD1

shRNA	Sequence (5'-3')
SCD1-Homo (shRNA#1)	CCGCTCTTACAAAGCTCGGCT
SCD1-Homo (shRNA#2)	GCACATCAACTTCACCACATT

Table S4. Short hairpin RNAs (shRNAs) sequence against MAFG

**shRNA**	**Sequence (5'-3')**
MAFG-Homo (shRNA#1)	GGGAAGGATTCACTCTCTTTA
MAFG-Homo (shRNA#2)	ACCTATTTGTGTGGTTATATA

Table S5. Primers used in qRT-PCR assays

Primer	Sequence (5'-3')
METTL14-Forward	GAACACAGAGCTTAAATCCCCA
METTL14-Reverse	TGTCAGCTAAACCTACATCCCTG
SCD1-Forward	TCTAGCTCCTATACCACCACCA
SCD1-Reverse	TCGTCTCCAACTTATCTCCTCC
β- actin-Forward	CATGTACGTTGCTATCCAGGC
β- actin -Reverse	CTCCTTAATGTCACGCACGAT

Table S6. Probes used in FISH assays

**Digoxin-labeled probe**	**Sequence (5'-3')**
SCD1	GCAAAGTGCGAGGAGTTGAC

Table S7. Primers used in MeRIP assays

Primer	Sequence (5'-3')
SCD1-Forward	AATTGCGTAGAGGCTACAGGG
SCD1-Reverse	CAGCAGACATTTCCAAGGGG
